# Analysis of government subsidy strategies in the supply chain of science and technology innovation platform

**DOI:** 10.1371/journal.pone.0323627

**Published:** 2025-05-16

**Authors:** Guangsi Zhang, Chen Chen, Jiaping Xie, Qiang Hu

**Affiliations:** 1 School of Business Administration, Xinjiang University of Finance and Economics, Urumqi, China; 2 College of Business, Shanghai University of Finance and Economics, Shanghai, China; 3 Hebei University of Water Resources and Electric Engineering, Cangzhou, China; 4 Digital Intelligence Management Research Institute, Shanghai University of Finance and Economics Zhejiang College, Jinhua, China; Fooyin University, TAIWAN

## Abstract

In the current era of technological advancements and intense global competition, innovation in Science and Technology (S&T) has become a crucial aspect of every country’s development strategy, and the development of cutting-edge technologies in core areas is of utmost importance. Innovation platforms that offer services for core technologies can be classified into three operational models: Public Welfare Platforms, Social Enterprise Platforms, and Commercial Platforms, depending on their objectives. To accelerate the realization of S&T innovation, the government provides subsidies to guide the S&T innovation platform and increase the participation of innovation subjects. With the help of optimization theory and supply chain theory, this paper constructed a model employing the game approach to delve into the pricing strategies of S&T platforms under three distinct models: Public Welfare Platform, Social Enterprise Platform, and Commercial Platform. The paper also discusses the effects of government subsidies on different innovation subjects. The study demonstrates that all three operational models can achieve the optimal membership fee for research users and the optimal commission rebate paid by the platform to resource providers. In the context of the Public Welfare Platform, despite the highest social welfare, the platform’s profit remains negative. Consequently, price subsidies are required for research users. At this juncture, the government subsidies to the S&T innovation platform yield the greatest social welfare yet the lowest profit for the platform. In contrast, the Social Enterprise Platform entails government subsidies for resource providers and research users, which can enhance the platform’s profit. The effect of subsidizing the resource providers on the profit growth of the S&T innovation platform is more significant than the effect on the improvement of social welfare. In the context of the Commercial Platform, regardless of the existence of government subsidies, the growth of social welfare and platform profits with the platform service level can be achieved. Furthermore, government subsidies for scientific research users are the most effective. These results provide theoretical and practical lessons for the pricing of the S&T innovation platform and the subject of government subsidies.

## 1 Introduction

In the current era of technological advancements and intense global competition, innovation in science and technology has become a crucial aspect of every country’s development strategy. Technological innovation has a positive role in sustainable process innovation [1]. Alliance activities between innovation agents significantly impact technological innovation [2]. As per available data, China possesses more scientific instruments and equipment than the combined total of 15 European Union countries. However, the utilization rate of these resources is less than 25%. Compared to developed countries, where instruments and equipment have a profit margin of 170%-200%, there needs to be more balance between the ownership and utilization of scientific and technological innovation resources in China^1^. This has resulted in China’s R&D expenditures increasing yearly, but the output of scientific and technological innovation could be better. To improve the utilization rate of S&T resources and accelerate their flow, the development of the S&T innovation platform has become crucial. These platforms serve as a bridge for fostering new, high-quality productivity and promoting S&T innovation. However, attracting S&T innovation resource providers and research users to join these platforms remains an urgent problem that needs to be solved.

To ensure a systematic flow of innovation factors, government subsidy support is essential in addition to the price means of the S&T innovation platform. This support is crucial for innovation subjects to concentrate on technology development and adhere to independent innovation. It is essential during the early stages of technological innovation to maximize social welfare [3]. The Chinese government has proposed accelerating the construction and optimization of science and technology resource-sharing service platforms. It aims to promote the opening and sharing of resources such as major scientific research infrastructures, large-scale scientific research instruments, and scientific and technological data through the dual-wheel drive of government guidance and market operation. However, innovation poses challenges such as significant investments, long return cycles, high R&D risks, and accessible knowledge spillovers [4]. Therefore, it is difficult for a single enterprise to achieve innovation in common technologies independently. To promote scientific and technological innovation, the government should subsidize the main bodies involved in innovation, such as resource providers, scientific research users, and the S&T innovation platform. To encourage innovation, the government has proposed several subsidy policies for innovators. For instance, the Beijing Municipal Science and Technology Commission and Zhongguancun Administrative Committee focused on the construction and development of national high-tech zones and the new round of early and pilot reforms in Zhongguancun, in line with the deployment requirements of the CPC Central Committee and State Council. They followed the laws of scientific and technological innovation. The Zhongguancun Demonstration Zone has released the “1+5” series of financial support policies aimed at supporting the development of high-precision industries and the construction of science and technology parks. These policies are based on the actual needs of innovation and entrepreneurial subjects. The five financial support policy documents have been put forward.

Other countries also have a range of subsidy strategies for Science and Technology innovation firms, but they do not break down Science and Technology innovation subjects and mainly subsidize specific areas. For example, the United States government, through the issuance of the Memorandum of Priorities for the Research and Development Budget and several relevant documents, has made it clear that areas such as artificial intelligence, quantum technology, and advanced communication network technology are the focus of future funding for scientific research and innovation. The German Federal government has implemented the ‘High-Tech Strategy 2020’, which focuses on and finances climate, energy, and communications. Japan’s Ministry of Economy, Trade and Industry (METI) has adjusted its subsidy policy for important material areas by the Economic Security Promotion Law, increasing support for key technology areas such as semiconductors, electronic components, and batteries.

China has correspondingly deployed key Science and Technology R&D areas, but in terms of subsidy policies, the main focus is on further segmentation of innovation agents.

In subsidizing S&T platforms, to incentivize innovative entities to actively undertake major national Science and Technology projects and create national Science and Technology innovation platforms, Fujian province of China has formulated and issued the Implementing Rules for Awarding and Subsidizing Major National Science and Technology Projects and National Science and Technology Innovation Platforms in 2023, detailing the subsidy policy for S&T platforms, and granting a one-off subsidy of RMB 5 million or RMB 3 million to eligible S&T platforms^2^. Sanya City of China has formulated the Measures for the Administration of Science and Technology Innovation and Entrepreneurship Platforms in Sanya Yazhou Bay Science and Technology City in 2024, which details the government’s subsidy criteria for S&T platforms and provides eligible S&T platforms with a one-off subsidy of RMB 1 million, RMB 800,000, RMB 300,000 and RMB 400,000, according to the requirements^3^. In terms of subsidizing resource providers, the Chinese government proposed in 2014 to provide subsidies to organizations that run scientific research instruments and equipment and formulated a series of policies. For example, the Administrative Measures for the Open Sharing of National Major Scientific Research Infrastructures and Large-Scale Scientific Research Instruments, introduced in 2017, proposes to provide government subsidies to eligible providers of Science and Technology innovation resources^4^. Under the guidance of the central policy, 26 provincial governments have formulated corresponding policies to offer subsidies to providers of Science and Technology innovation resources^5^. For example, the Fujian Provincial Department of Science and Technology has formulated the 2024 Pilot Programmed for Subsidies for Enterprises’ Large-Scale Research Instruments to Open Services to the Community, which provides one-time subsidies to enterprises that offer research instruments^6^. In terms of research users, to promote the integration of Science and Technology innovation resources and enhance the incentives for enterprises to engage in Science and Technology innovation, 2024 Sichuan province of China has released a policy on incentives and subsidies for Science and Technology-based small and medium-sized enterprises (SMEs) in all cities and states. For example, Dujiangyan City of China gives a one-off subsidy of RMB 10,000 to Science and Technology-based SMEs joining the platform, and Ya’an City of China gives subsidy support of up to RMB 1 million to Science and Technology-based SMEs carrying out technological innovations based on the technological level of their research projects and the expected economic and social benefits^7^.

Furthermore, the S&T innovation platform that provides linkage services for S&T innovation must maintain a balance between public good and commercial attributes to ensure their long-term viability. Innovation platforms can be classified into three operational models based on different goals. The first is the Public Welfare Platform, which aims to maximize social welfare. The second is the Commercial Platform, which aims to maximize the platform’s profit. The last one is the Social Enterprise Platform, which considers both the public welfare and commercial attributes. To fully stimulate the innovation initiative of the S&T innovation platform and accelerate the flow of S&T resources, it is important to urgently study how the S&T innovation platform formulates pricing strategies and how the government chooses the main body of subsidies.

Therefore, to better address the issue of S&T innovation resource flow and analyze the optimal strategy for government subsidy effectiveness, this paper considers three types of innovation entities: resource providers, scientific research users, and S&T innovation platforms. It applies optimization theory, supply chain theory, and game theory to conduct the research. The main research questions are: how do platforms determine the optimal pricing strategy under three different operation models, and which type of innovation entity does government subsidy have the best effect on? Different from previous studies, this paper innovatively presents the pricing strategy for S&T innovation platforms and, for the first time, compares and analyzes the effect of government subsidies on S&T innovation entities. This not only enriches the theoretical research in the field of operation and management but also provides theoretical references for S&T innovation activities, making it a valuable contribution to theoretical and academic research.

## 2 Literature review

This paper covers research in three areas: Platform pricing strategy, government subsidies, and market-oriented operation of enterprises with public good attributes. Scholars have extensively researched platform pricing and have reached a consensus that optimal pricing in bilateral platforms is influenced by network externalities rather than being solely determined by demand elasticity or marginal cost [5,6]. Additionally, it has been found that platforms can maximize their profits in networked markets by charging reasonable fees to the bilaterals [7,8]. The issue of bilateral pricing for platforms can be approached from various angles. Zhang et al. [9] suggested that a fixed-fee contract charging commissions is more effective in improving product quality on the platform. Martínez-de-Albéniz et al. [10] studied the bilateral pricing problem by considering suppliers’ inventories and selling seasons and found that dynamic commission charges to suppliers benefit the long-term development of e-commerce platforms. Tan et al. [11] analyzed the bilateral pricing strategy from a platform integration perspective. They found that the platform can attract bilateral users by enhancing platform integration and improving service quality. Among others, past research on platform pricing strategies had predominantly centered on e-commerce platforms [12,13], rental service platforms [14,15], and content platforms [16,17]. While existing studies have examined platform pricing decisions from various angles, there were fewer studies focused on the S&T innovation platform that carried a certain level of innovation risk, and the limited studies that existed were primarily based on empirical research methods [18,19], providing inadequate exploration of platform pricing strategies within these platforms. Therefore, this paper employed optimization theory and game-theoretic approaches to delve into the platform pricing problem of the S&T platform from a microscopic perspective.

In studies related to platforms in the past, two types of subsidies were generally considered, with platforms having subsidized users [20] or governments having subsidized platforms or users [21]. Government subsidies can improve the innovation environment and motivate innovation subjects. Government support could have provided favorable conditions for users to engage in innovative activities while simultaneously contributing to the enhancement of social welfare [22]. The pricing strategies of platforms can also be affected by government subsidies [23]. Research has shown that government subsidies target both consumers and firms [3,24]. Subsidizing consumers can promote the adoption of innovative technologies [25,26]. According to research, state subsidies could enhance the commercial viability of startups [27]. Additionally, Conti [28] discovered that R&D subsidies have a significant positive impact on the survival of startups, their ability to attract external investment, and their innovation, using data from startups in a foreign country. Furthermore, Zhou et al. [29] demonstrated through an evolutionary game model that the strength of government subsidies to firms affects the incentives of firms to join the platform. Previous studies have often considered innovation to be the responsibility of producers. However, it has been found that as the proportion of demand-side user innovation increases, government subsidies for producers to innovate will decrease social welfare [22]. Existing studies on government subsidies have not yet encompassed a comparative analysis of the three operational models of the S&T platform. Consequently, this paper undertakes a comparative analysis of the impact that government subsidies exert on three distinct types of innovation subjects within the context of these three operational models of the S&T platform.

Social enterprises, which are entities that operate between public welfare organizations and commercial enterprises, adopt a market-oriented operational model similar to that of commercial enterprises. This allows them to achieve their sustainable development while pursuing the maximization of social value [30]. Their commercial goal is to achieve a certain economic profit, while their social goal is to maximize social welfare. Their commercial goal is to achieve a certain economic profit, while their social goal is to maximize social welfare. Resource-sharing platforms typically serve the public interest sector. Therefore, it is important to study the operation model of such platforms from the regulator’s perspective [31]. Benjaafar et al. [32] compared the equilibrium results of consumers’ willingness to own products, product usage efficiency, and social welfare in sharing platforms under profit maximization and social welfare maximization, respectively. They found that the difference in social welfare obtained from maximizing profit and maximizing social welfare for decision analysis was relatively small. However, they did not analyze the profit problem of platforms under social welfare maximization. Jung et al. [33] analyzed the optimal pricing and innovative service quality of platforms under different subsidy policies from the perspective of government policymaking. They aimed to maximize platform profit while also considering social welfare. Although research on platforms had been conducted by scholars who considered both platform revenue and social welfare, neither platform revenue nor social welfare was factored into the model. Drawing upon the research of social enterprises, this paper formulated a platform pricing model tailored to the operational mode of social enterprises and incorporated both platform income and social welfare into its considerations.

Many scholars have already researched the pricing of platforms, the provision of government subsidies, and the market-oriented operation of enterprises with public welfare attributed from a variety of theoretical and methodological perspectives. However, no literature has considered the pricing of S&T innovation platforms, their operation models with public welfare attributes, and the comparative analysis of the impacts of different government subsidy strategies on social welfare and platform profits. In this paper, three distinct platform operation models were depicted, each aiming to maximize either platform revenue, social welfare, or social welfare while also striving for platform profit maximization. The paper subsequently determined platform pricing under these three modes and conducted a comparative analysis of the impact of government subsidies under various operational models. Based on this analysis, the article offers both theoretical and practical insights into the pricing and government subsidy strategies for S&T platforms.

## 3 Basic model

**Fig 1:**
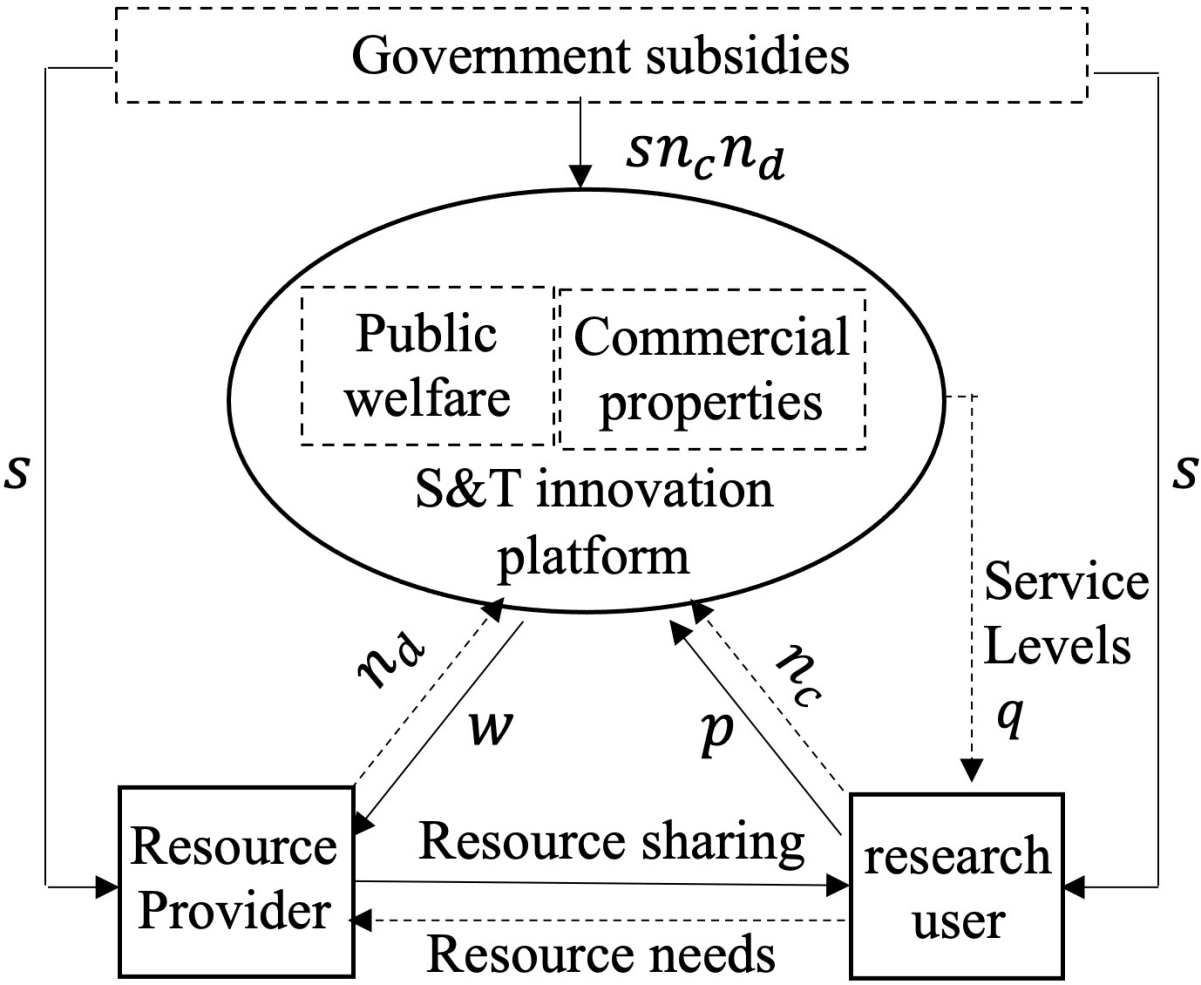
Structure of S&T innovation platform pricing and government subsidies.

The research object of this paper includes three types of innovation subjects: resource providers, research users and S&T innovation platforms. S&T innovation platform offers a one-time commission rebate (denoted by w) to resource providers to incentivize them to join the platform and provide S&T resources. Additionally, they provide innovation services at a quality level (denoted by *q*) and charge a one-time membership fee (denoted by p) to attract research users to join the platform. To accelerate the promotion of S&T innovation, the government provides subsidies to the three innovation actors, resource providers, research users, and S&T innovation platform, to increase their motivation towards S&T innovation initiatives. The government provides a one-time subsidy (denoted by s) to each resource provider and research user, as well as a unit subsidy to the S&T innovation platform based on the transaction volume. This paper constructed a pricing model for S&T platforms under three operation models using optimization theory and game methods. This paper considers the public welfare attribute and commercial attribute of the S&T innovation platform. It constructs a pricing model for the S&T innovation platform under three operational models: a public welfare platform pricing model that maximizes social welfare, a social enterprise platform pricing model that considers both social welfare and platform profit and a commercial platform pricing model that maximizes platform profit. It also considers the effect of government subsidies based on the three pricing models. The specific platform pricing and government subsidy structure model is depicted in Fig 1, and the model symbols and meanings are presented in Table 1.

**Table 1:** Notations and descriptions for the model.

Type	Notations	Descriptions	Notations	Descriptions
**Parameters**	$\alpha_{c}$	Coefficient of cross-side network externalities for research users	$\alpha_{d}$	Coefficient of cross-side network externalities for resource provider
	$\theta_{c}$	The retained utility of research users joining the platform	$f_{d}$	The cost of effort to be paid by resource providers to join the platform
	q	The level of quality of services provided by the Platform to research users	$s_{g}$	Unit subsidy provided by the Government
	η	The probability of failure of scientific and technological innovation by scientific research users	μ	The negative utility of failure to research users
	ρ	The cost factor of the implementation of innovative services by the science and technology innovation platform		
**Decision variables**	p	Fixed membership fee charged by the platform to research users	w	Commission paid by the S&T innovation platform to S&T resource providers
**Functions**	$U_{c}$	The utility gained by research users joining the platform	$n_{c}$	Number of research users opting into the platform
	$U_{d}$	The utility gained by a resource provider joining the platform	$n_{d}$	Number of innovation resource providers opting into the platform
	$\pi_{p}$	Profit from S&T innovation platform	SW	Social welfare

This study assumes that each resource provider on the platform offers only one unit of S&T innovation resources, and each research user can only access one unit of S&T innovation resources. Under the base model, the utility obtained by each resource provider joining the platform can be expressed as. When $U_{d}\geq 0$, i.e.,$\;f_{d}\leq \alpha_{d}n_{c}+w$, the effort cost paid by the resource provider to join the platform is less than the critical value ${\tilde{f}}_{d}=\alpha_{d}n_{c}+w$, the provider will choose to join the platform. It is assumed that the distribution of $f_{d}$, the effort cost paid by each resource provider for joining the platform, is the same and uniformly distributed on [0,1]. Thus, the formula to express the number of resource providers joining the S&T innovation platform is $n_{d}=\int_{0}^{{\tilde{f}}_{d}}d\left(f_{d}\right)={\tilde{f}}_{d}=\alpha_{d}n_{c}+w$. Referring to Tan et al.‘s research hypothesis [34], it is assumed that the probability of scientific research users failing to carry out scientific and technological innovations on the S&T innovation platform is η (0<η<1). The negative utility that failure will bring to the scientific research users is μ. To ensure practical significance, let 0<μ<1. Then the utility obtained by each scientific research user joining the platform can be expressed as $U_{c}=\theta_{c}+\alpha_{c}n_{d}+q-p-\mu \eta$, the retained utility of the scientific research user joining the platform is greater than the critical value of ${\tilde{\theta }}_{c}=p+\mu \eta -\alpha_{c}n_{d}-q$ will choose to join the platform, also assuming that each scientific research user joining the platform to obtain the same distribution of retained utility $\theta_{c}$ distribution, are [0,1] on the uniform distribution.Therefore, the number of scientific research users willing to join the platform, denoted as $n_{c}=\int_{{\tilde{\theta }}_{c}}^{1}d\left(\theta_{c}\right)=1-{\tilde{\theta }}_{c}=1+\alpha_{c}n_{d}+q-p-\mu \eta$. Solving the expressions $n_{d}$ and $n_{c}$ simultaneously can be obtained as follows:


$$n_{c}=\frac{1-p+q-\eta \mu +w\alpha_{c}}{1-\alpha_{c}\alpha_{d}}$$ (1)

$$n_{d}=\frac{w+\alpha_{d}(1-p+q-\eta \mu )}{1-\alpha_{c}\alpha_{d}}$$ (2)

Residuals for resource providers and research users can be expressed as $DS=\int_{0}^{{\tilde{f}}_{d}}\left(U_{d}\right)d\left(f_{d}\right)=\int_{0}^{\alpha_{d}n_{c}+w}\left(\alpha_{d}n_{c}+w-f_{d}\right)d\left(f_{d}\right)$ and $CS=\int_{{\tilde{\theta }}_{c}}^{1}\left(U_{c}\right)d\left(\theta_{c}\right)=\int_{p+\eta \mu -\alpha_{c}n_{d}-q}^{1}\left(\theta_{c}+\alpha_{c}n_{d}+q-p-\eta \mu \right)d\left(\theta_{c}\right)$ respectively, and profits for S&T innovation platform can be expressed as $\pi_{p}^{ns}=pn_{c}-wn_{d}-\frac{1}{2}\rho q^{2}$.

### 3.1 Public welfare platform

Under the Public Welfare Platform, the S&T innovation platform fully plays its public welfare attributes to maximize social welfare as a goal, using the superscript a to denote the Public Welfare Platform. The social welfare SW can be expressed as three components, i.e., the surplus of resource providers, the surplus of research users, and the profit of the S&T innovation platform, i.e., ${SW}^{a}={DS}^{a}+{CS}^{a}+\pi_{p}^{a}$. Under this objective, the S&T innovation platform decides the membership price p charged to the research users and the commission rebate w paid to the resource providers at the same time, so the maximization problem of the Public Welfare Platform can be expressed as follows:


$$\begin{array}{c} \max{SW}^{a}\\ s.t.\;n_{c}^{a}>0,\;n_{d}^{a}>0 \end{array}$$ (3)

**Proposition 1**. If $0<\alpha_{c}+\alpha_{d}<1$, there is a unique equilibrium solution ($p^{a^{*}},w^{a^{*}}$) under the Public Welfare Platform model of operation that achieves the goal of maximizing social welfare [See A.1 for proof details].

Proposition 1 demonstrates that optimal user fees and commission rebates exist that maximize social welfare when there is no government subsidy and the sum of cross-side network externalities of platform users is not large.

### 3.2 Social enterprise platform

The Social Enterprise Platform operates intending to maximize social welfare and platform profit. To fully stimulate the market operation vitality of the S&T innovation platform, the profit of the platform should be taken into consideration while pursuing the maximization of social welfare. This study applies Stackelberg game theory to depict the master-slave game model for maximizing social welfare and the profit of the S&T innovation platform under the operation model of a Social Enterprise Platform. The order of the game is as follows: the S&T innovation platform first decides to charge research users to maximize social welfare and then decides to pay commission rebates w to resource providers to maximize platform profit. The operation model of the Social Enterprise Platform is indicated by the superscript b. Social welfare SW is composed of three components: resource provider surplus, research user surplus, and S&T innovation platform profit, i.e., $\;{SW}^{b}={DS}^{b}+{CS}^{b}+\pi_{p}^{b}$. Thus, the maximization problem for the Social Enterprise Platform can be expressed as follows:


$$\begin{array}{c} \max{SW}^{b}\\ s.t.\max\pi_{p}^{b}\\ n_{c}^{b}>0,n_{d}^{b}>0 \end{array}$$ (4)

**Proposition 2**. There is a unique equilibrium solution ($p^{b^{*}},\;w^{b^{*}}$) under the operation model of the Social Enterprise Platform that can maximize social welfare while also maximizing the profit of the S&T innovation platform [see A.2 for proof details].

Proposition 2 shows that in the case of no government subsidy, there exists an optimal user fee and commission rebate that maximizes social welfare while maximizing the profit of the S&T innovation platform.

### 3.3 Commercial platform

The Commercial Platform aims to maximize the profit of the S&T innovation platform from a commercial perspective. This involves determining the membership price p charged to research users and the commission rebate w paid to resource providers. The superscript c denotes the enterprise-led operation model. Thus, the maximization problem under the Commercial Platform can be expressed as:


$$\begin{array}{c} \max\pi_{p}^{c}\\ s.t.\;n_{c}^{c}>0,n_{d}^{c}>0 \end{array}$$ (5)

**Proposition 3**. There is a unique equilibrium solution ($p^{c^{*}},w^{c^{*}}$) under the Commercial Platform that can achieve the profit maximization objective of the S&T innovation platform [See A.3 for proof details].

Proposition 3 shows that there is an optimal user fee and commission rebate that can maximize the profit of the S&T innovation platform without government subsidy.

**Property 1**. From Proposition 1 to Proposition 3, we can derive the variation of the optimal price $p^{*}$ charged by the S&T innovation platform to research users and the commission rebate $w^{*}$ paid to resource providers with the platform’s service level q under the three operational models of Public Welfare Platform, Social Enterprise Platform, and Commercial Platform. [See A.4 for the proof details]

(1) The operation model of the Public Welfare Platform is as follows:$\;\frac{\partial p^{a^{*}}}{\partial q}<0,\;\frac{\partial w^{a^{*}}}{\partial q}>0$.
(2) The operation model of the Social Enterprise Platform is as follows: $\frac{\partial p^{b^{*}}}{\partial q}>0,\;$if $\alpha_{c}>2\alpha_{d}$, and $\frac{\partial w^{b^{*}}}{\partial q}<0$, if $\alpha_{d}>\alpha_{c}$.
(3) The operation model of the commercial platform is as follows: $\frac{\partial p^{c^{*}}}{\partial q}>0$, $\frac{\partial w^{c^{*}}}{\partial q}>0$ if $\alpha_{d}<\alpha_{c}$, and $\frac{\partial w^{c^{*}}}{\partial q}<0$ if $\alpha_{d}>\alpha_{c}$.

Property 1 Illustration: If the service level of the S&T innovation platform improves, the fees for research users and commission rebates for resource providers will change under the three operation models:

(1) When operating under the Public Welfare Platform model, it is recommended to reduce the fees charged to research users and increase the commission rebates paid to resource providers.
(2) when operating under the Social Enterprise Platform model, it is suggested to increase user fees if research users are more sensitive to cross-side network effects and to decrease commission rebates to resource providers if they are more sensitive to cross-side network effects.
(3) In the Commercial Platform, fees for research users may be increased. If research users are more sensitive to the cross-side network effect, the commission rebate for resource providers should be increased. Conversely, if resource providers are more sensitive to the cross-side network effect, the commission rebate for them should be reduced.

## 4 Government subsidy strategy

National policy guidance and support are important for innovation subjects to focus on technology development and adhere to independent innovation. Government subsidies for science and technology innovation fields that promote social development are of great significance, especially in the early stages of innovation. Government subsidies have a certain impact on the development of science and technology innovation platform service supply chains.

### 4.1 Description of resource provider and research user sizes

#### 4.1.1 Subsidize resource provider.

When subsidizing resource providers, their utility for joining the platform can be expressed as $U_{d}^{sd}=\alpha_{d}n_{c}^{sd}+w+s_{g}-f_{d}$, which is the same as the base model. Providers will choose to join the platform if their utility is positive. The number of providers willing to join the platform can be expressed as $n_{d}^{sd}=\int_{0}^{{\tilde{f}}_{d}^{sd}}d\left(f_{d}\right)={\tilde{f}}_{d}^{sd}=\alpha_{d}n_{c}^{sd}+w+s_{g}$. The utility gained by each research user joining the platform is the same as that of the base model, expressed as $U_{c}^{sd}=U_{c}^{b}$. The number of research users willing to join the platform can be expressed as $n_{c}^{sd}=\int_{{\tilde{\theta }}_{c}^{sd}}^{1}d\left(\theta_{c}\right)=1-{\tilde{\theta }}_{c}^{sd}=1+\alpha_{c}n_{d}^{sd}+q-p-\mu \eta$. The solution is obtained by associating the $n_{d}^{sd}$ and $n_{c}^{sd}$ expressions:


$$n_{c}^{sd}=\frac{1-p+q-\eta \mu +\alpha_{c}(w+s_{g})}{1-\alpha_{c}\alpha_{d}}$$ (6)

$$n_{d}^{sd}=\frac{w+s_{g}+\alpha_{d}(1-p+q-\eta \mu )}{1-\alpha_{c}\alpha_{d}}$$ (7)

#### 4.1.2 Subsidize research user.

In the case of government-subsidized research users, the utility obtained by each research user upon joining the platform can be expressed as $U_{c}^{sc}=\theta_{c}+\alpha_{c}n_{d}^{sc}+q+s_{g}-p-\eta \mu$, which is the same as the base model. Research users will choose to join the platform if the utility is positive, and the number of research users willing to join the platform is $n_{c}^{sc}=\int_{{\tilde{\theta }}_{c}^{sc}}^{1}d\left(\theta_{c}\right)=1-{\tilde{\theta }}_{c}^{sc}=1+\alpha_{c}n_{d}^{sc}+q+s_{g}-p-\eta \mu$. The platform offers the same utility to each resource provider as in the base model, which is $U_{d}^{sc}=U_{d}^{b}$. The number of providers willing to join the platform is $n_{d}^{sc}=\int_{0}^{{\tilde{f}}_{d}^{sc}}d\left(f_{d}\right)={\tilde{f}}_{d}^{sc}=\alpha_{d}n_{c}^{sc}+w$. The solution is obtained by associating the $n_{c}^{sc}$ and $n_{d}^{sc}$ expressions:


$$n_{c}^{sc}=\frac{1-p+q-\eta \mu +s_{g}+w\alpha_{c}}{1-\alpha_{c}\alpha_{d}}$$ (8)

$$n_{d}^{sc}=\frac{w+\alpha_{d}(1-p+q-\eta \mu +s_{g})}{1-\alpha_{c}\alpha_{d}}$$ (9)

#### 4.1.3 Subsidize S&T innovation platform.

According to Rochet and Tirole [5], government subsidies to platforms are often linked to the volume of transactions on the platform. The volume of transactions on the platform can be denoted by $n_{c}n_{d}$. Therefore, the platform’s profit under government subsidy can be expressed as $\pi_{p}^{sp}=pn_{c}^{sp}+s_{g}n_{c}^{sp}n_{d}^{sp}-wn_{d}^{sp}-\frac{1}{2}\rho q^{2}$. The utility expression for resource providers and research users joining the platform under the scenario of government subsidy is the same as the basic model. The expressions for the number of resource providers and research users joining the platform and their residuals are also the same as those of the basic model, i.e., $n_{c}^{sp}=n_{c}$, $n_{d}^{sp}=n_{d}$.

### 4.2 Public welfare platform

#### 4.2.1 Subsidize resource provider.

Under the Public Welfare Platform, the surplus of resource providers and research users, after government subsidies, can be represented as ${DS}^{sda}=\int_{0}^{{\tilde{f}}_{d}^{sda}}\left(U_{d}^{sda}\right)d\left(f_{d}\right)=\int_{0}^{\alpha_{d}n_{c}^{sda}+w+s_{g}}\left(\alpha_{d}n_{c}^{sda}+w+s_{g}-f_{d}\right)d\left(f_{d}\right)$ and ${CS}^{sda}=\int_{{\tilde{\theta }}_{c}^{sda}}^{1}\left(U_{c}^{sda}\right)d\left(\theta_{c}\right)=\int_{p+\mu \eta -\alpha_{c}n_{d}^{sda}-q}^{1}\left(\theta_{c}+\alpha_{c}n_{d}^{sda}+q-p-\mu \eta \right)d\left(\theta_{c}\right)$ respectively. The profit of the S&T innovation platform can be expressed as $\pi_{p}^{sda}=pn_{c}^{sda}-wn_{d}^{sda}-\frac{1}{2}\rho q^{2}$, and the social welfare as ${SW}^{sda}={DS}^{sda}+{CS}^{sda}+\pi_{p}^{sda}$. Therefore, the maximization problem of subsidizing resource providers under the Public Welfare Platform can be expressed as follows:


$$\begin{array}{c} \max{SW}^{sda}\\ s.t.n_{c}^{sda}>0,n_{d}^{sda}>0 \end{array}$$ (10)

**Proposition 4**. If $0<\alpha_{c}+\alpha_{d}<1$, a unique equilibrium solution ($p^{sda^{*}},w^{sda^{*}}$) exists for government subsidies to resource providers under the Public Welfare Platform model of operation. This solution maximizes social welfare. [See A.5 for proof details]

Proposition 4 demonstrates that when the sum of cross-side network externalities of platform users is not significant under the Public Welfare Platform, there exists an optimal user fee and commission rebate that maximizes social welfare, provided that the government subsidizes the resource provider.

#### 4.2.2 Subsidize research user.

Under the operation model of the Public Welfare Platform, in which the government subsidizes research users, the surplus of resource providers and research users can be expressed as ${DS}^{sca}=\int_{0}^{{\tilde{f}}_{d}^{sca}}\left(U_{d}^{sca}\right)d\left(f_{d}\right)=\int_{0}^{\alpha_{d}n_{c}^{sca}+w}\left(\alpha_{d}n_{c}^{sca}+w-f_{d}\right)d\left(f_{d}\right)$ and ${CS}^{sca}=\int_{{\tilde{\theta }}_{c}^{sca}}^{1}\left(U_{c}^{sca}\right)d\left(\theta_{c}\right)=\int_{p+\eta \mu -\alpha_{c}n_{d}^{sca}-q-s_{g}}^{1}\left(\theta_{c}+\alpha_{c}n_{d}^{sca}+q+s_{g}-p-\eta \mu \right)d\left(\theta_{c}\right)$, respectively. The profit of the S&T innovation platform can be expressed as $\pi_{p}^{sca}=pn_{c}^{sca}-wn_{d}^{sca}-\frac{1}{2}\rho q^{2}$, and the social welfare can be expressed as ${SW}^{sca}={DS}^{sca}+{CS}^{sca}+\pi_{p}^{sca}$. The maximization problem of the operation model of the Public Welfare Platform under the subsidy of research users can be expressed as follows:


$$\begin{array}{c} \max{SW}^{sca}\\ s.t.\;n_{c}^{sca}>0,\;n_{d}^{sca}>0 \end{array}$$ (11)

**Proposition 5**. If $0<\alpha_{c}+\alpha_{d}<1$, a unique equilibrium solution ($p^{{sca}^{*}},w^{{sca}^{*}}$) exists for government subsidies to scientific research users under the Public Welfare Platform model of operation. This solution can help the S&T innovation platform achieve the goal of maximizing social welfare. [See A.6 for proof details]

Proposition 5 demonstrates that when the sum of cross-side network externalities of platform users is not significant, government subsidies to research users are necessary to maximize social welfare with optimal user fees and commission rebates under the Public Welfare Platform.

#### 4.2.3 Subsidize S&T innovation platform.

When the government provides subsidies for the S&T innovation platform, the utility of resource providers and research users remains the same as when there is no subsidy, i.e.,${\;DS}^{sp}=DS$, ${CS}^{sp}=CS$. Social welfare can be expressed as ${SW}^{sp}={DS}^{sp}+{CS}^{sp}+\pi_{p}^{sp}$. In the Public Welfare Platform, the problem of maximizing government subsidies for the S&T innovation platform can be expressed as:


$$\begin{array}{c} \max{SW}^{spa}\\ s.t.\;n_{c}^{spa}>0,\;n_{d}^{spa}>0 \end{array}\;$$ (12)

**Proposition 6**. If $0<\alpha_{c}+\alpha_{d}+s_{g}<1$, a unique equilibrium solution ($p^{{spa}^{*}},w^{{spa}^{*}}$) exists for government subsidies to the S&T innovation platform under the Public Welfare Platform model of operation. This solution maximizes social welfare. [See A.7 for proof details]

Proposition 6 demonstrates that when the sum of cross-side network externalities of platform users is not significant, there exists an optimal user fee and commission rebate for government subsidies to S&T innovation platform to maximize social welfare under the operation model of Public Welfare Platform.

**Property 2**. Propositions 4–6 demonstrate that, when operating under the Public Welfare Platform model, the government provides subsidies to three types of innovation entities. The optimal price $p^{*}$ charged by the S&T innovation platform to research users and the commission rebate $w^{*}$ paid to resource providers vary based on the platform’s service level q and the government subsidy $s_{g}$. [refer to A.8 for proof details]

(1) Government-subsidized resource providers:$\;\frac{\partial p^{{sda}^{*}}}{\partial q}<0$,$\;\frac{\partial w^{sda^{*}}}{\partial q}>0$, $\frac{\partial p^{{sda}^{*}}}{\partial s_{g}}<0$,$\;\frac{\partial w^{sda^{*}}}{\partial s_{g}}>0$.
(2) Government-subsidized research users:$\;\frac{\partial p^{{sca}^{*}}}{\partial q}<0$,$\;\frac{\partial w^{sca^{*}}}{\partial q}>0$,$\;\frac{\partial p^{{sca}^{*}}}{\partial s_{g}}<0$,$\;\frac{\partial w^{sca^{*}}}{\partial s_{g}}>0$.
(3) Government-subsidized innovation platform:$\;\frac{\partial p^{{spa}^{*}}}{\partial q}<0$,$\;\frac{\partial w^{spa^{*}}}{\partial q}>0$,$\;\frac{\partial p^{{spa}^{*}}}{\partial s_{g}}<0$,$\;\frac{\partial w^{spa^{*}}}{\partial s_{g}}>0$.

Property 2 Illustration: under the Public Welfare Platform, the government subsidizes the three types of innovation subjects enough to get the same trend, and the trend is consistent with that in the absence of government subsidies.

(1) If the S&T innovation platform implements a higher level of service, it should reduce the membership fee charged to S&T users and increase the commission rebate paid to resource providers.
(2) If the government increases subsidies, the S&T innovation platform should reduce the membership fees charged to S&T users and increase the commission rebates paid to resource providers.

### 4.3 Social enterprise platform

#### 4.3.1 Subsidize resource provider.

The government subsidy resource provider situation under the operation model of Social Enterprise is modeled using the Stackelberg game model to maximize social welfare and profit of S&T innovation platform. The model can be expressed as follows:


$$\begin{array}{c} \max{SW}^{sdb}\\ s.t.\;\max\pi_{p}^{sdb}\\ n_{c}^{sdb}>0,\;n_{d}^{sdb}>0 \end{array}$$ (13)

**Proposition 7**. There is a single equilibrium solution $\left(p^{{sdb}^{*}},w^{{sdb}^{*}}\right)$ for the government-subsidized resource provider under the Social Enterprise Platform. This solution satisfies the premise of maximizing the profit of the S&T innovation platform to achieve the maximum social welfare. [See A.9 for proof details].

Proposition 7 demonstrates that, under the Social Enterprise Platform, the government subsidizes resource providers through the availability of optimal user fees and commission rebates, allowing the S&T innovation platform to maximize social welfare while also maximizing profit.

#### 4.3.2 Subsidize research user.

In the Social Enterprise operation model, we establish a Stackelberg game model that maximizes social welfare and S&T innovation platform profit when the government subsidizes scientific research users. The model can be expressed as follows:


$$\begin{array}{c} \max{SW}^{scb}\\ s.t.\;\max\pi_{p}^{scb}\\ n_{c}^{scb}>0,\;n_{d}^{scb}>0 \end{array}$$ (14)

**Proposition 8**. The existence of a unique equilibrium solution ($p^{{scb}^{*}}$, $w^{{scb}^{*}}$) for government-subsidized research users under the Social Enterprise Platform can satisfy the maximization of social welfare while also maximizing the profit of the S&T innovation platform [See A.10 for proof details].

Proposition 8 reveals that, under the Social Enterprise Platform, the government subsidizes research users through optimal user fees and commission rebates to maximize social welfare and the profit of the S&T innovation platform.

#### 4.3.3 Subsidize S&T innovation platform.

The Stackelberg game model of maximizing social welfare and maximizing profit of S&T innovation platform is constructed under the Social Enterprise Platform’s operating model for the scenario where S&T innovation platform is being subsidized by the government. This can be represented as follows:


$$\begin{array}{c} \max{SW}^{spb}\\ .t.\;\max\pi_{p}^{spb}\\ n_{c}^{spb}>0,\;n_{d}^{spb}>0 \end{array}\;$$ (15)

**Proposition 9**. The government-subsidized S&T innovation platform under the Social Enterprise Platform has a unique equilibrium solution ($p^{{spb}^{*}},w^{{spb}^{*}}$) that maximizes social welfare while satisfying the profit maximization of the S&T innovation platform [See A.11 for proof details].

Proposition 9 demonstrates that there is an optimal user fee and commission rebate for government subsidies to S&T innovation platform under the operation model of Social Enterprise Platform. This approach maximizes social welfare while also maximizing the profit of S&T innovation platform.

**Property 3**. From Proposition 7 to Proposition 9, it can be inferred that the government subsidizes all three types of innovation subjects under the Social Enterprise Platform. The optimal price $p^{*}$ charged by the S&T innovation platform to research users and the commission rebate $w^{*}$ paid to resource providers vary with the platform’s service level q and the government’s subsidy $s_{g}\;$[See A.12 for proof details].

(1) Government-subsidized resource providers: $\frac{\partial w^{{sdb}^{*}}}{\partial s_{g}}<0$, if `$\alpha_{c}>2\alpha_{d}$,$\;\frac{\partial p^{{sdb}^{*}}}{\partial q}>0$; if $\alpha_{d}>\alpha_{c}$, $\frac{\partial w^{{sdb}^{*}}}{\partial q}<0$, $\frac{\partial p^{{sdb}^{*}}}{\partial s_{g}}<0$.
(2) Government-subsidized research users: if $\alpha_{c}>2\alpha_{d}$, $\frac{\partial p^{{scb}^{*}}}{\partial q}>0$, $\frac{\partial p^{{scb}^{*}}}{\partial s_{g}}>0$; if $\alpha_{d}>\alpha_{c}$, $\frac{\partial w^{{scb}^{*}}}{\partial q}<0$, $\frac{\partial w^{{scb}^{*}}}{\partial s_{g}}<0$.

Property 3 Illustration: the operation model of the Social Enterprise Platform, where the government provides enough subsidies to resource providers and scientific research users to bring about the following change trend:

(1) With the increase in the level of services implemented by the S&T innovation platform, if the research users are more sensitive to the cross-side network effect, their membership fees will be increased, and if the resource providers are more sensitive to the cross-side network effect, the commission rebates paid to them should be reduced.
(2) With the increase of government subsidy, if the government subsidizes the resource providers, the S&T innovation platform should reduce the commission rebate paid to them, and at this time, for the scientific research users who are more sensitive to the cross-side network effect, the membership fee should be lowered; if the government subsidizes the scientific research users, the membership fee should be raised if the scientific research users are more sensitive to the cross-side network effect, and the commission rebate paid to the resource providers should be lowered if the resource providers are more sensitive to the cross-side network effect.

### 4.4 Commercial platform

#### 4.4.1 Subsidize resource provider.

The Commercial Platform operates with the profit maximization objective of the S&T innovation platform from a commercial perspective. The S&T innovation platform decides on the membership price p to be charged to research users and the commission rebate w to be paid to resource providers. Thus, the maximization problem for the operation model of the Commercial Platform, when resource providers are subsidized, can be expressed as:


$$\begin{array}{c} \max\pi_{p}^{sdc}\\ s.t.n_{c}^{sdc}>0,\;n_{d}^{sdc}>0 \end{array}$$ (16)

**Proposition 10.** There is a single equilibrium solution ($p^{{sdc}^{*}},w^{{sdc}^{*}}$) that maximizes the profitability of the S&T innovation platform under the Commercial Platform, where the resource providers are subsidized by the government [See A.13 for proof details].

Proposition 10 demonstrates that in the Commercial Platform, where the government subsidizes the resource provider, optimal user fees and commission rebates exist, making the S&T innovation platform the most profitable.

#### 4.4.2 Subsidize research user.

In the case of subsidizing scientific research users, the maximization problem of the Commercial Platform can be expressed as follows:


$$\begin{array}{c} \max\pi_{p}^{scc}\\ s.t.\;n_{c}^{scc}>0,\;n_{d}^{scc}>0 \end{array}$$ (17)

**Proposition 11**. There is a single equilibrium solution ($p^{{scc}^{*}},w^{{scc}^{*}}$) for government-subsidized research users under the Commercial Platform. This solution maximizes the profit of the S&T innovation platform. [See A.14 for proof details]

Proposition 11 demonstrates that, under the Commercial Platform, an optimal user fee and commission rebate for the government to subsidize research users exists, which maximizes the profit of the S&T innovation platform.

#### 4.4.3 Subsidize S&T innovation platform.

The maximization problem of the Commercial Platform in the case of subsidized S&T innovation platform can be expressed as follows:


$$\begin{array}{c} \max\pi_{p}^{spc}\\ s.t.\;n_{c}^{spc}>0,\;n_{d}^{spc}>0 \end{array}$$ (18)

**Proposition 12**. The Commercial Platform is characterized by a unique equilibrium solution ($p^{{spc}^{*}},w^{{spc}^{*}}$) for the government-subsidized S&T innovation platform that maximizes the profitability of the S&T innovation platform [See A.15 for proof details].

Proposition 12 demonstrates that under the Commercial Platform, the government subsidizes the S&T innovation platform, resulting in an optimal user fee and commission rebate that maximizes the S&T innovation platform’s profit.

**Property 4**. From Proposition 10 to Proposition 12, it can be inferred that the government subsidizes three types of innovation subjects under the operation model of the Social Enterprise Platform. The optimal price $p^{*}$ charged by the S&T innovation platform to research users and the commission rebate $w^{*}$ paid to resource providers vary with the service level q of the platform and the government subsidy $s_{g}\;$[See A.16 for proof details].

(1) Government-subsidized resource providers: $\;\frac{\partial p^{{sdc}^{*}}}{\partial q}>0$, $\frac{\partial w^{sdc^{*}}}{\partial s_{g}}<0$. If $\alpha_{c}<\alpha_{d}$,$\;\frac{\partial w^{{sdc}^{*}}}{\partial q}<0$,$\;\frac{\partial p^{{sdc}^{*}}}{\partial s_{g}}<0$; if $\alpha_{c}>\alpha_{d}$,$\;\frac{\partial w^{sdc^{*}}}{\partial q}>0$,$\;\frac{\partial p^{{sdc}^{*}}}{\partial s_{g}}>0$.
(2) Government-subsidized research users: $\frac{\partial p^{{scc}^{*}}}{\partial q}>0$,$\;\frac{\partial p^{{scc}^{*}}}{\partial s_{g}}>0$. If $\alpha_{c}<\alpha_{d}$,$\;\frac{\partial w^{scc^{*}}}{\partial q}<0$,$\;\frac{\partial w^{scc^{*}}}{\partial s_{g}}<0$; if $\alpha_{c}>\alpha_{d}$,$\;\frac{\partial w^{scc^{*}}}{\partial q}>0$,$\;\frac{\partial w^{scc^{*}}}{\partial s_{g}}>0$.
(3) Government-subsidized S&T innovation platform: $\;\frac{\partial p^{{spc}^{*}}}{\partial q}>0$,$\;\frac{\partial p^{{spc}^{*}}}{\partial s_{g}}<0$,$\;\frac{\partial w^{spc^{*}}}{\partial s_{g}}>0$. If $\alpha_{d}<\alpha_{c}+s_{g}$,$\;\frac{\partial w^{spc^{*}}}{\partial q}>0$; if $\alpha_{d}>\alpha_{c}+s_{g}$,$\;\frac{\partial w^{spc^{*}}}{\partial q}<0$.

Property 4 Illustration: under the Commercial Platform, the government provides subsidies to the three types of innovation subjects to induce a certain change trend.

(1) As the level of services provided by the S&T innovation platform increases, regardless of the type of S&T innovation main body that is subsidized by the government, the S&T innovation platform should raise the membership fee for scientific research users. Furthermore, the commission rebate should be reduced for resource providers if they are more sensitive to the cross-side network effect, and conversely, increased for resource providers if scientific research users are more sensitive to the cross-side network effect.
(2) When the government provides subsidies, the platform should adjust its policies accordingly. Specifically, the commission rebate for resource providers should be reduced, while the membership fee for S&T innovation platform should be increased for research users. On the other hand, when the government subsidizes the S&T innovation platform, the membership fee for research users should be reduced, and the commission rebate for resource providers should be increased. Additionally, the platform should consider reducing the membership fee for research users or the commission rebate for resource providers when the resource providers are more sensitive to the cross-side network effect. Conversely, if research users demonstrate higher sensitivity to the cross-side network effect, the platform should raise the membership fee for research users or increase the commission rebate for resource providers.

A summary of Properties 1–4 yields Table 2, which in turn yields Conclusion 1, Conclusion 2, and Conclusion 3.

**Table 2:** Analysis of equilibrium solutions under three models of operation.

	Public Welfare Platform				Social Enterprise Platform				Commercial Platform			
	$\frac{\partial p^{*}}{\partial 𝐪}$	$\frac{\partial w^{*}}{\partial 𝐪}$	$\frac{\partial p^{*}}{\partial s_{g}}$	$\frac{\partial w^{*}}{\partial s_{g}}$	$\frac{\partial p^{*}}{\partial 𝐪}$	$\frac{\partial w^{*}}{\partial 𝐪}$	$\frac{\partial p^{*}}{\partial s_{g}}$	$\frac{\partial w^{*}}{\partial s_{g}}$	$\frac{\partial p^{*}}{\partial 𝐪}$	$\frac{\partial w^{*}}{\partial 𝐪}$	$\frac{\partial p^{*}}{\partial s_{g}}$	$\frac{\partial w^{*}}{\partial s_{g}}$
ns	–	+	/	/	+ ^③^	–^④^	/	/	–	–^①^	/	/
sd	–	–	–	+	+ ^③^	–^④^	–^④^	–	+	± ^①^	± ^①^	–
sc	–	+	–	+	+ ^③^	–^④^	+ ^③^	–^④^	+	± ^①^	+	± ^①^
sp	–	+	–	+	/	/	/	/	+	± ^②^	–	+

ns: no government subsidies; sd: government-subsidized resource providers; sc:government-subsidized research users; sp: government-subsidized S&T innovation platform;

① $\frac{\partial w^{*}}{\partial q}>0$,$\frac{\partial p^{*}}{\partial s_{g}}>0$,$\frac{\partial w^{*}}{\partial s_{g}}>0$,if $\alpha_{d}<\alpha_{c}$; $\frac{\partial w^{*}}{\partial q}<0$,$\frac{\partial p^{*}}{\partial s_{g}}<0$,$\frac{\partial w^{*}}{\partial s_{g}}<0$,if $\alpha_{d}>\alpha_{c}$;

② $\frac{\partial w^{*}}{\partial q}>0$,if $\alpha_{d}<\alpha_{c}+s_{g}$; $\frac{\partial w^{*}}{\partial q}<0$, if $\alpha_{d}>\alpha_{c}+s_{g}$;

③$\frac{\partial p^{*}}{\partial q}>0$,$\frac{\partial p^{*}}{\partial s_{g}}>0$,if $\alpha_{c}>2\alpha_{d}$;

④ $\frac{\partial w^{*}}{\partial q}<0$,$\frac{\partial p^{*}}{\partial s_{g}}<0$,if $\alpha_{d}>\alpha_{c}$.

**Conclusion 1**. When operating under the Public Welfare Platform model, the S&T innovation platform should lower fees for research users and increase commission rebates for resource providers in order to maximize social welfare. This should be done regardless of whether government subsidies are available.

**Conclusion 2**. When operating under the Social Enterprise Platform model, the S&T innovation platform should increase the membership fee for research users who are more sensitive to the cross-side network effect and reduce the commission rebate for resource providers who are more sensitive to the cross-side network effect. This will increase the profit of the S&T innovation platform.

**Conclusion 3**. When operating under the Commercial Platform model, the S&T innovation platform should increase the membership fee of research users to maximize the platform’s profit when providing high-level services, regardless of government subsidies. Additionally, if resource providers are more sensitive to the cross-side network effect, they should lower their commission rebates, and vice versa.

## 5 Equilibrium comparative analysis

### 5.1 Comparison of three platform models without government subsidy

From Proposition 1 to Proposition 3, we can ascertain the optimal membership fees charged by the platforms to research users and the commission rebates paid to resource providers under the three operation models of Public Welfare Platform, Social Enterprise Platform, and Commercial Platform when there is no government subsidy. As illustrated in Table 3.

**Table 3:** Equilibrium solutions under the three models of operation.

	$p^{*}$	$w^{*}$
**Public Welfare Platform(**a)	$-\frac{\alpha_{d}(1+q-\eta \mu )(\alpha_{c}+\alpha_{d})}{1-{\left(\alpha_{c}+\alpha_{d}\right)}^{2}}$	$\frac{\alpha_{c}(1+q-\eta \mu )}{1-{\left(\alpha_{c}+\alpha_{d}\right)}^{2}}$
**Social Enterprise Platform(**b)	$\frac{(1+q-\eta \mu )\left(\alpha_{c}+\alpha_{d}\right)\left(3\alpha_{d}-\alpha_{c}\left(2-\alpha_{d}\left(\alpha_{c}-2\alpha_{d}\right)\right)\right)}{2\left(\alpha_{c}\alpha_{d}\left(3-\alpha_{d}^{2}\right)-2\right)-\left(1-\alpha_{c}^{2}\right)\left(\alpha_{c}^{2}-3\alpha_{d}^{2}\right)}$	$\frac{\left(1+q-\eta \mu )(2\alpha_{d}-\alpha_{c}\left(\alpha_{c}^{2}+\alpha_{d}^{2}\right)\right)}{2\left(\alpha_{c}\alpha_{d}\left(3-\alpha_{d}^{2}\right)-2\right)-\left(1-\alpha_{c}^{2}\right)\left(\alpha_{c}^{2}-3\alpha_{d}^{2}\right)}$
**Commercial Platform(**c)	$\frac{(1+q-\eta \mu )\left(2-\alpha_{d}\left(\alpha_{c}+\alpha_{d}\right)\right)}{4-{\left(\alpha_{c}+\alpha_{d}\right)}^{2}}$	$\frac{\left(1+q-\eta \mu )(\alpha_{c}-\alpha_{d}\right)}{4-{\left(\alpha_{c}+\alpha_{d}\right)}^{2}}\backslash )$

By performing calculations and comparisons outlined in Table 3, we have determined the most favorable membership price $p^{c^{*}}>p^{b^{*}}>p^{a^{*}}$, and the optimal commission rebate that platforms should pay to resource suppliers, denoted as $w^{b^{*}}<w^{c^{*}}<w^{a^{*}}$, under the three platform operation models without government subsidies. These findings support Conclusion 4. [See A.17 for proof details].

**Conclusion 4**. The Commercial Platform has the highest membership fee charged by the S&T innovation platform to research users, while the Social Enterprise Platform has the lowest commission rebate paid by the S&T innovation platform to resource providers. In other words, without government subsidies, high-income regions should adopt the Commercial Platform operating model in which the platform raises fees for research users. Instead, low-income regions should adopt the Public Welfare Platform operating model where the platform reduces the fees charged to research users.

### 5.2 Comparison of subsidy strategies under public welfare platform

From Proposition 1 and Propositions 4–6, we can determine the optimal membership fee charged by the platform to research users and the optimal commission rebate paid to resource providers under the Public Welfare Platform where the government subsidizes resource providers, research users, and the S&T innovation platform, respectively. The results are presented in Table 4.

**Table 4:** Equilibrium solutions for different subsidy strategies under the public welfare platform.

	$p^{*}$	$w^{*}$
a	$-\frac{\alpha_{d}(1+q-\eta \mu )(\alpha_{c}+\alpha_{d})}{1-{\left(\alpha_{c}+\alpha_{d}\right)}^{2}}$	$\frac{\alpha_{c}(1+q-\eta \mu )}{1-{\left(\alpha_{c}+\alpha_{d}\right)}^{2}}$
sda	$-\frac{\alpha_{d}\left(s_{g}+(1+q-\eta \mu )\left(\alpha_{c}+\alpha_{d}\right)\right)}{1-{\left(\alpha_{c}+\alpha_{d}\right)}^{2}}$	$\frac{\alpha_{c}\left(1+q-\eta \mu +s_{g}\left(\alpha_{c}+\alpha_{d}\right)\right)}{1-{\left(\alpha_{c}+\alpha_{d}\right)}^{2}}$
sca	$-\frac{\alpha_{d}(\alpha_{c}+\alpha_{d})(1+q-\eta \mu +s_{g})}{1-{\left(\alpha_{c}+\alpha_{d}\right)}^{2}}$	$\frac{\alpha_{c}(1+q-\eta \mu +s_{g})}{1-{\left(\alpha_{c}+\alpha_{d}\right)}^{2}}$
spa	$-\frac{\left(\alpha_{d}+s_{g}\right)(1+q-\eta \mu )(\alpha_{c}+\alpha_{d}+s_{g})}{1-{\left(\alpha_{c}+\alpha_{d}+s_{g}\right)}^{2}}$	$\frac{\left(\alpha_{c}+s_{g}\right)(1+q-\eta \mu )}{1-{\left(\alpha_{c}+\alpha_{d}+s_{g}\right)}^{2}}\backslash )$

A computational comparison of the research user fees $p^{spa^{*}}<p^{sda^{*}}<p^{sca^{*}}<p^{a^{*}}<0$ and commission rebates $w^{spa^{*}}>w^{sca^{*}}>w^{sda^{*}}>w^{a^{*}}>0$ paid to resource providers under different government subsidy strategies under the Public Welfare Platform can be obtained from the calculations in Table 4, which leads to Conclusion 5. [See A.18 for proof details]

**Conclusion 5**. In the context of the Public Welfare Platform, the S&T innovation platform must subsidize the price to scientific research users and pay commission rebates to resource providers. The platform should be implemented most strongly when the government subsidizes the S&T innovation platform. Under this mode of operation, because the platform already provides a reverse income subsidy to research users, the government of the high-income region may no longer subsidize the innovation subjects, and the government of the low-income region may provide a subsidy to the S&T innovation platform.

The calculation also yields that under the Public Welfare operating model, if $\alpha_{d}>\frac{1}{2}$, when there is no government subsidy, $\left|p^{a^{*}}\right|>w^{a^{*}}$, when the government subsidizes the resource provider, $\left|p^{sda^{*}}\right|>w^{sda^{*}}$, when the government subsidizes the research user, $\left|p^{sda^{*}}\right|>w^{sda^{*}}$, when the government subsidizes the S&T innovation platform, when $\alpha_{d}>\frac{1-s_{g}}{2}$, $\left|p^{spa^{*}}\right|>w^{spa^{*}}$. This leads to Conclusion 6. [See A.19 for proof details].

**Conclusion 6**. In the context of the Public Welfare Platform, it can be posited that if the resource providers are more sensitive to network effects, the S&T innovation platform should provide more price subsidies to research users.

### 5.3 Comparison of subsidy strategies under commercial platform

From Proposition 3 and Propositions 10–12, we can determine the optimal membership fee charged by the platform to research users and the optimal commission rebate paid to resource providers under the Commercial Platform where the government subsidizes resource providers, research users, and the S&T innovation platform, respectively. The results are presented in Table 5.

**Table 5:** Equilibrium solutions for different subsidization strategies of commercial platform.

	$p^{*}$	$w^{*}$
c	$\frac{(1+q-\eta \mu )\left(2-\alpha_{d}\left(\alpha_{c}+\alpha_{d}\right)\right)}{4-{\left(\alpha_{c}+\alpha_{d}\right)}^{2}}$	$\frac{\left(1+q-\eta \mu )(\alpha_{c}-\alpha_{d}\right)}{4-{\left(\alpha_{c}+\alpha_{d}\right)}^{2}}$
sdc	$\frac{s_{g}\left(\alpha_{c}-\alpha_{d}\right)+(1+q-\eta \mu )\left(2-\alpha_{d}\left(\alpha_{c}+\alpha_{d}\right)\right)}{4-{\left(\alpha_{c}+\alpha_{d}\right)}^{2}}$	$\frac{(1+q-\eta \mu )\left(\alpha_{c}-\alpha_{d}\right)-s_{g}\left(2-\alpha_{c}\left(\alpha_{c}+\alpha_{d}\right)\right)}{4-{\left(\alpha_{c}+\alpha_{d}\right)}^{2}}$
scc	$\frac{\left(1+q-\eta \mu +s_{g}\right)\left(2-\alpha_{d}\left(\alpha_{c}+\alpha_{d}\right)\right)}{4-{\left(\alpha_{c}+\alpha_{d}\right)}^{2}}$	$\frac{\left(1+q-\eta \mu +s_{g})(\alpha_{c}-\alpha_{d}\right)}{4-{\left(\alpha_{c}+\alpha_{d}\right)}^{2}}$
spc	$\frac{(1+q-\eta \mu )\left(2-\left(\alpha_{d}+s_{g}\right)\left(\alpha_{c}+\alpha_{d}+s_{g}\right)\right)}{4-{\left(\alpha_{c}+\alpha_{d}+s_{g}\right)}^{2}}$	$\frac{\left(1+q-\eta \mu )(\alpha_{c}-\alpha_{d}+s_{g}\right)}{4-{\left(\alpha_{c}+\alpha_{d}+s_{g}\right)}^{2}}\backslash )$

A comparison of the various government subsidy scenarios under the Commercial Platform operating model, as calculated in Table 5, reveals the following [See A.20 for proof details]:

(1) $p^{c^{*}}>0$, $p^{scc^{*}}>0$, $p^{sdc^{*}}>0$ if $\alpha_{c}>\alpha_{d}$, $p^{sdc^{*}}<0$ if $\alpha_{c}<\alpha_{d}$ and $\begin{array}{c} s_{g}>\frac{(1+q-\eta \mu )\left(2-\alpha_{d}\left(\alpha_{c}+\alpha_{d}\right)\right)}{\alpha_{d}-\alpha_{c}} \end{array}$; $p^{spc^{*}}>0$ if $0<\alpha_{c}<\frac{2-{\left(\alpha_{d}+s_{g}\right)}^{2}}{\alpha_{d}+s_{g}}$, $p^{spc^{*}}<0$ if $\alpha_{c}>\frac{2-{\left(\alpha_{d}+s_{g}\right)}^{2}}{\alpha_{d}+s_{g}}$;
(2) $p^{scc^{*}}>p^{sdc^{*}}>p^{c^{*}}>p^{spc^{*}}$ if $\alpha_{c}>\alpha_{d}$, $p^{scc^{*}}>p^{c^{*}}>p^{sdc^{*}}>p^{spc^{*}}$ if $\alpha_{c}<\alpha_{d}$;
(3) When $\alpha_{d}<\alpha_{c}$ then $w^{c^{*}}>0$,$w^{scc^{*}}>0$,$w^{spc^{*}}>0$, thereinto $w^{sdc^{*}}>0$ if $\begin{array}{c} s_{g}<\frac{(1+q-\eta \mu )\left(\alpha_{c}-\alpha_{d}\right)}{2-\alpha_{c}\left(\alpha_{c}+\alpha_{d}\right)} \end{array}$, $w^{sdc^{*}}<0$ if $\begin{array}{c} s_{g}>\frac{(1+q-\eta \mu )\left(\alpha_{c}-\alpha_{d}\right)}{2-\alpha_{c}\left(\alpha_{c}+\alpha_{d}\right)} \end{array}$, When $\alpha_{d}>\alpha_{c}$ then $w^{c^{*}}<0$, $w^{scc^{*}}<0$, $w^{sdc^{*}}<0$, thereinto $w^{spc^{*}}<0$ if $s_{g}<\alpha_{d}-\alpha_{c}$; $w^{spc^{*}}>0$ if $s_{g}>\alpha_{d}-\alpha_{c}$;
(4) $w^{c^{*}}>w^{spc^{*}}>w^{scc^{*}}>w^{{sdc}^{*}}$ if $\alpha_{c}<\alpha_{d}$.

**Conclusion 7**. In the model of operation of commercial platforms, if the resource provider is more sensitive to the cross-border network effect and the government subsidizes it more, the platform should provide price subsidies to the scientific research users to attract scientific research users to join the platform, and if the scientific research users are sensitive to the cross-border network effect, the platform should provide price subsidies to the scientific research users when the government subsidizes the scientific and technological innovation platform. Under this general operating model, governments in high-income areas should subsidize resource providers, and governments in low-income areas should subsidize the S&T innovation platform.

**Conclusion 8**. In the context of the Commercial Platform, even if research users are more sensitive to the cross-side network effect if the government subsidizes resource providers to a significant extent, the platform should apply an inverse pricing structure to resource providers. Conversely, if resource providers are more sensitive to the cross-side network effect, the platform should apply an inverse pricing structure to resource providers when the government subsidizes research users and subsidizes resource providers, and when it subsidizes the S&T innovation platform.

## 6 Numerical simulation analysis

In this section, by portraying important parameters and conducting numerical example simulation, we can intuitively understand the impact of the service level of the S&T innovation platform and the intensity of government subsidies under different operation models and government subsidies for different innovation subjects and compare the platform profits and social welfare under different operation models and different government subsidy strategies. Basic parameters $\alpha_{c}=0.3$, $\alpha_{d}=0.2$, ρ=0.8, μ=0.5, η=0.5.

### 6.1 Impact of service level

#### 6.1.1 Basic model.

**Fig 2:**
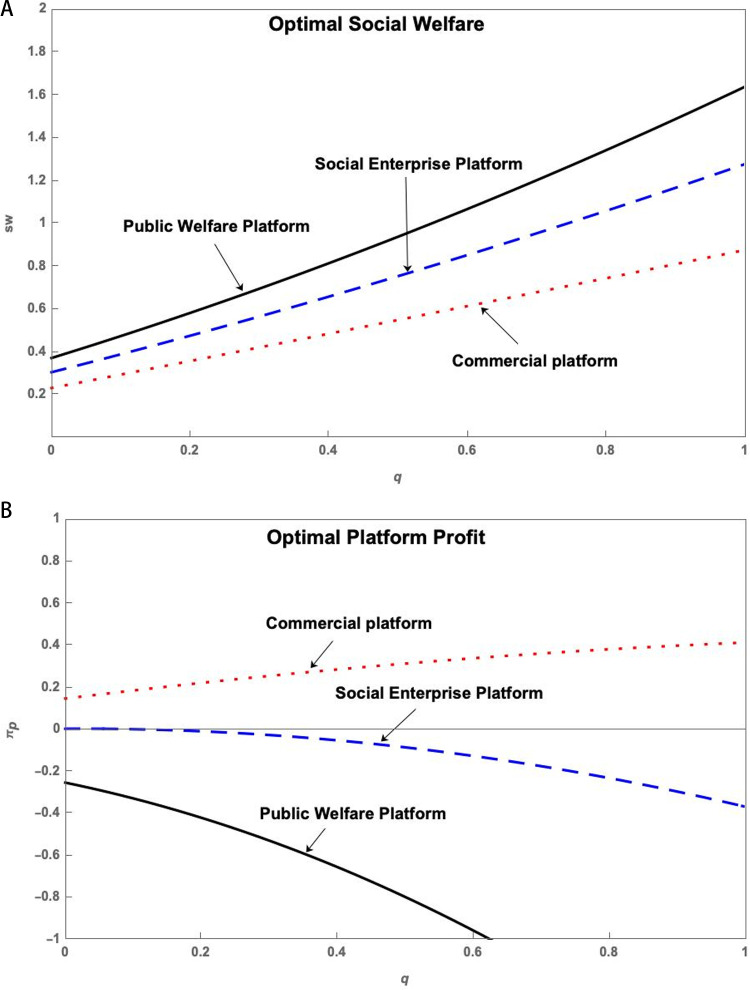
(a) The impact of the q on ${sw}^{*}$ of three platforms. (b) The impact of the q on $\pi_{p}^{*}$ of three platforms.

Under the three operation models of Public Welfare Platform, Social Enterprise Platform, and Commercial Platform, through simulation, the impact of the service level q of the S&T innovation platform on the social welfare ${sw}^{*}$ and the profit $\pi_{p}^{*}$ of the S&T innovation platform can be obtained, as shown in Fig 2. (The simulation with multiple sets of data can yield the same trend of change.)

Fig 2 shows that social welfare increases as platform service level increases in all three models of operation. However, platform profit decreases significantly with the increase in platform service level in the Public Welfare Platform model of operation and increases significantly in the Commercial Platform model of operation. Among the three types of innovative entities, the Public Welfare Platform can achieve the highest social welfare but the lowest platform profit, while the Commercial Platform can obtain the highest platform profit but has the lowest social welfare. The Social Enterprise Platform falls in between, bringing both social welfare and platform profit.

#### 6.1.2 Government subsidy.

To ascertain the impact of the service level of the S&T innovation platform on social welfare and platform profits under the scenario of government subsidies to the three types of innovation subjects, the government subsidy strategies under the operation models of Public Welfare Platform, Social Enterprise Platform, and Commercial Platform are simulated. thereinto the government subsidies $s_{g}=0.2$. (The simulation with multiple sets of data can yield the same trend of change.)

(1) Public Welfare Platform

**Fig 3:**
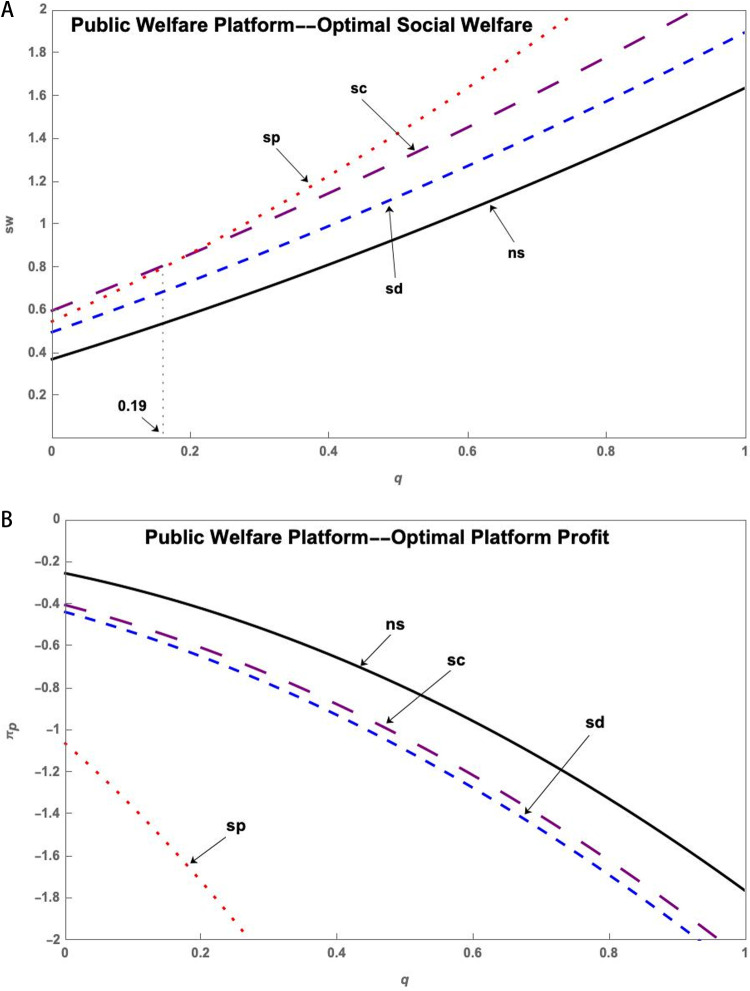
(a) The impact of the q on ${sw}^{*}$ of Public Welfare Platform under government subsidy. (b) The impact of the q on $\pi_{p}^{*}$ of Public Welfare Platform under government subsidy.

As shown in Fig 3, government subsidies for innovation subjects under the Public Welfare Platform do not affect the optimal social welfare and platform profits. Subsidies for the S&T innovation platform can increase social welfare but result in lower platform profits. This phenomenon becomes more apparent with higher service levels of the S&T innovation platform. Subsidizing research users can increase social welfare, while also allowing the S&T innovation platform to generate higher profits.

(2) Social Enterprise Platform

**Fig 4:**
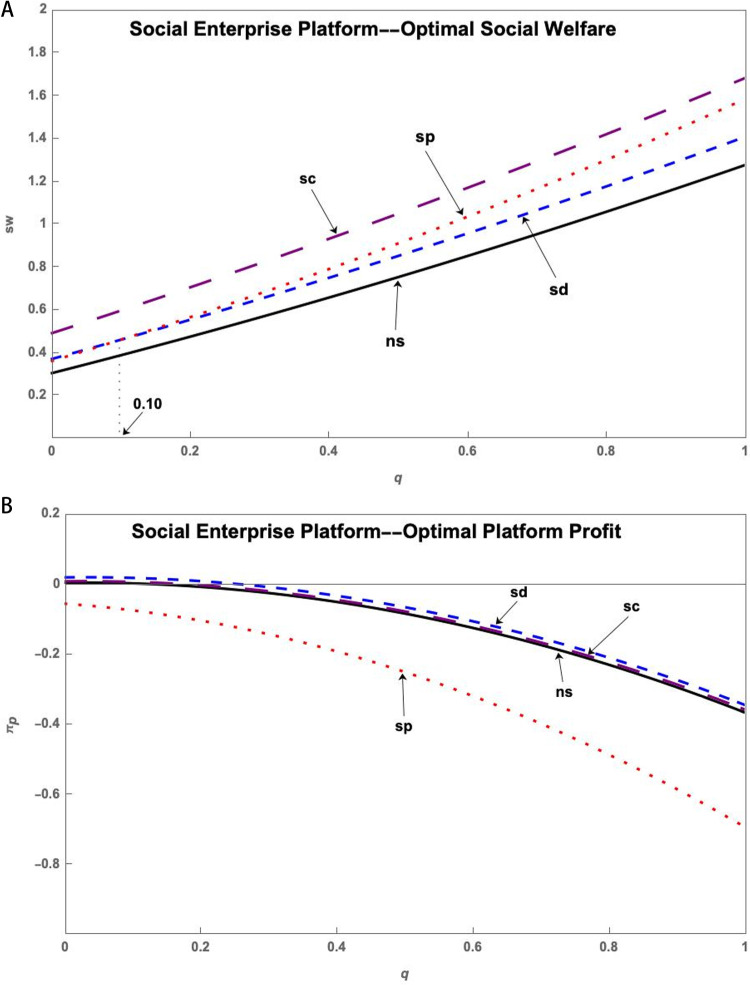
(a) The impact of the q on ${sw}^{*}$ of Social Enterprise Platform under government subsidy. (b) The impact of the q on $\pi_{p}^{*}$ of Social Enterprise Platform under government subsidy.

Fig 4 shows that the government subsidizes research users to achieve the highest social welfare under the Social Enterprise Platform. At this point, the government does not subsidize or subsidize research users and resource providers to bring similar profits to the platform. The platform has the lowest profits when subsidizing the S&T innovation platform.

(3) Commercial Platform

**Fig 5:**
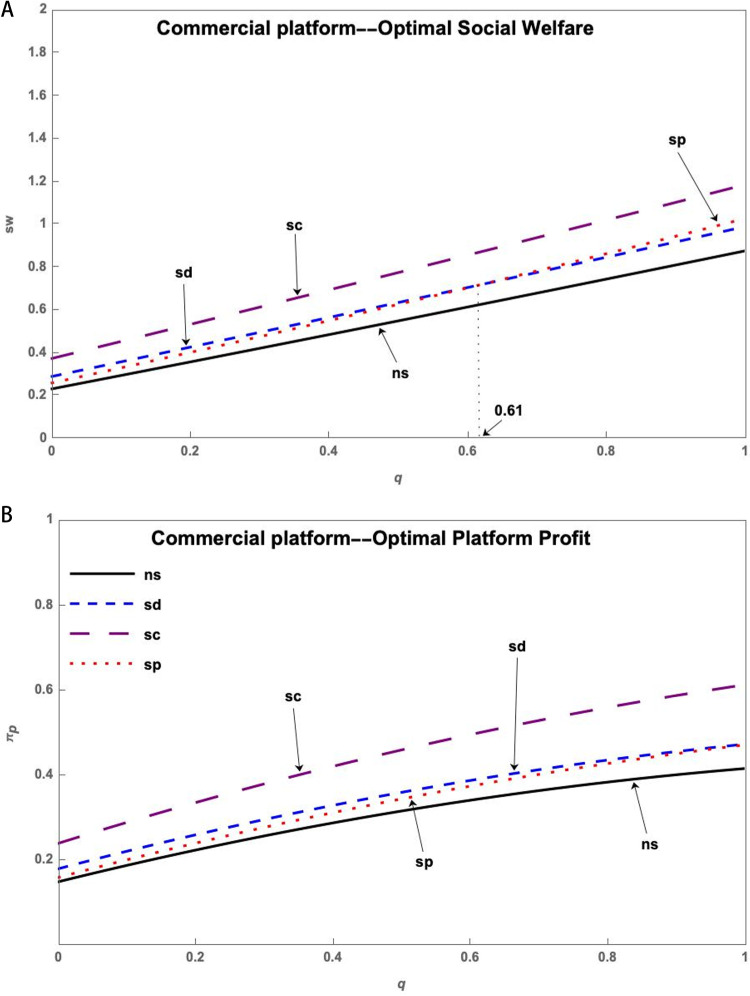
(a) The impact of the q on ${sw}^{*}$ of Commercial Platform under government subsidy. (b) The impact of the q on $\pi_{p}^{*}$ of Commercial Platform under government subsidy.

Fig 5 demonstrates that government subsidies to research users in the Commercial Platform operating model can result in the highest social welfare and platform profits.

**Conclusion 9**. In the Commercial Platform, government subsidies to research users can increase both social welfare and platform profits, with both increasing as the platform service level increases.

### 6.2 Impact of subsidy strength

To ascertain the impact of government subsidies on social welfare and platform profits under the scenarios of government subsidies to the three types of innovative entities, the government subsidy strategies under the operation models of Public Welfare Platform, Social Enterprise Platform, and Commercial Platform are simulated, respectively, in which the government subsidies are q=0.5 (the simulation with multiple sets of data can yield the same trend of change).

(1) Public Welfare Platform

**Fig 6:**
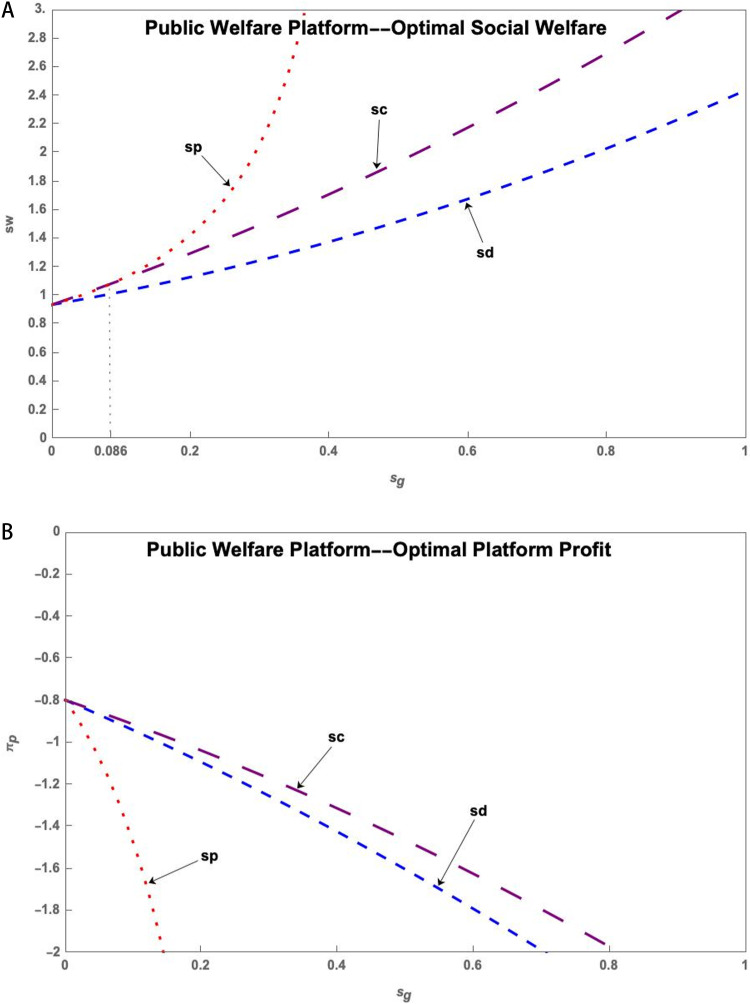
(a) The impact of the $s_{g}$ on ${sw}^{*}$ of Public Welfare Platform. (b) The impact of the $s_{g}$ on $\pi_{p}^{*}$ of Public Welfare Platform.

Fig 6 illustrates that the government provides subsidies to the S&T innovation platform under the Public Welfare Platform to increase social welfare. The social welfare increases with the increase of the subsidy, but the S&T innovation platform has the lowest profit at this point. Providing subsidies to research users can also increase social welfare and enable the S&T innovation platform to obtain higher profits.

(2) Social Enterprise Platform

**Fig 7:**
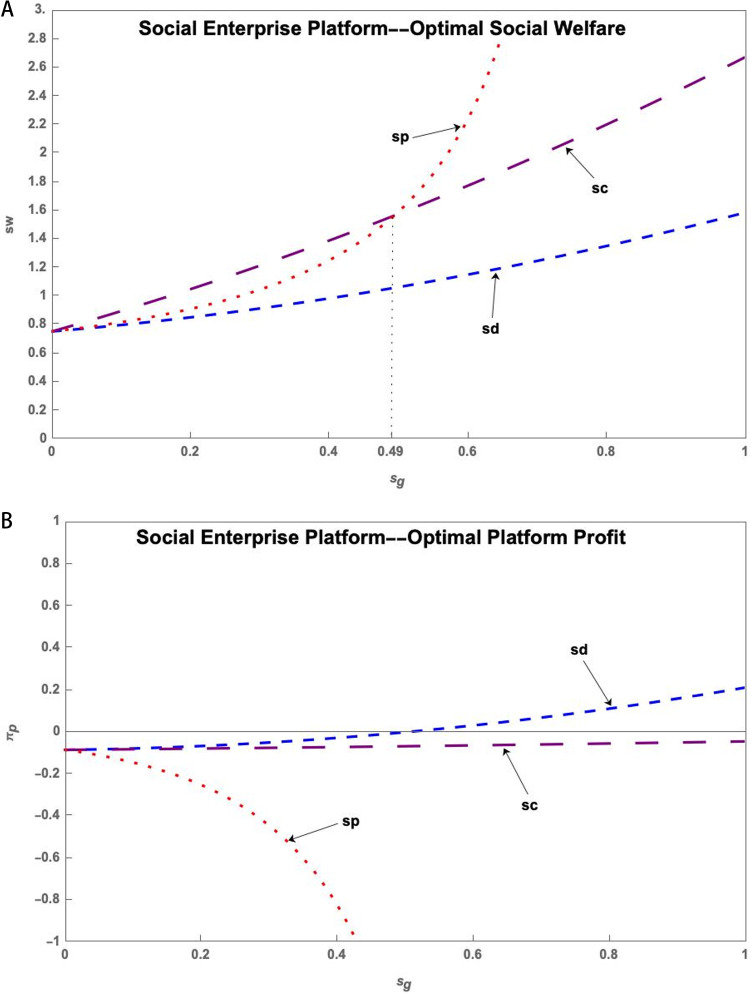
(a) The impact of the $s_{g}$ on ${sw}^{*}$ of Social Enterprise Platform. (b) The impact of the $s_{g}$ on $\pi_{p}^{*}$ of Social Enterprise Platform.

Fig 7 shows that under the Social Enterprise Platform, the government subsidizes research users to obtain the highest social welfare when the subsidy is not too strong. The S&T innovation platform needs to be heavily subsidized to obtain higher social welfare, but the subsidized S&T innovation platform has the lowest profit, which decreases with the increase of the subsidy. At this point, the government subsidizes the research users and the resource providers to bring higher profits to the platform.

(3) Commercial Platform

**Fig 8:**
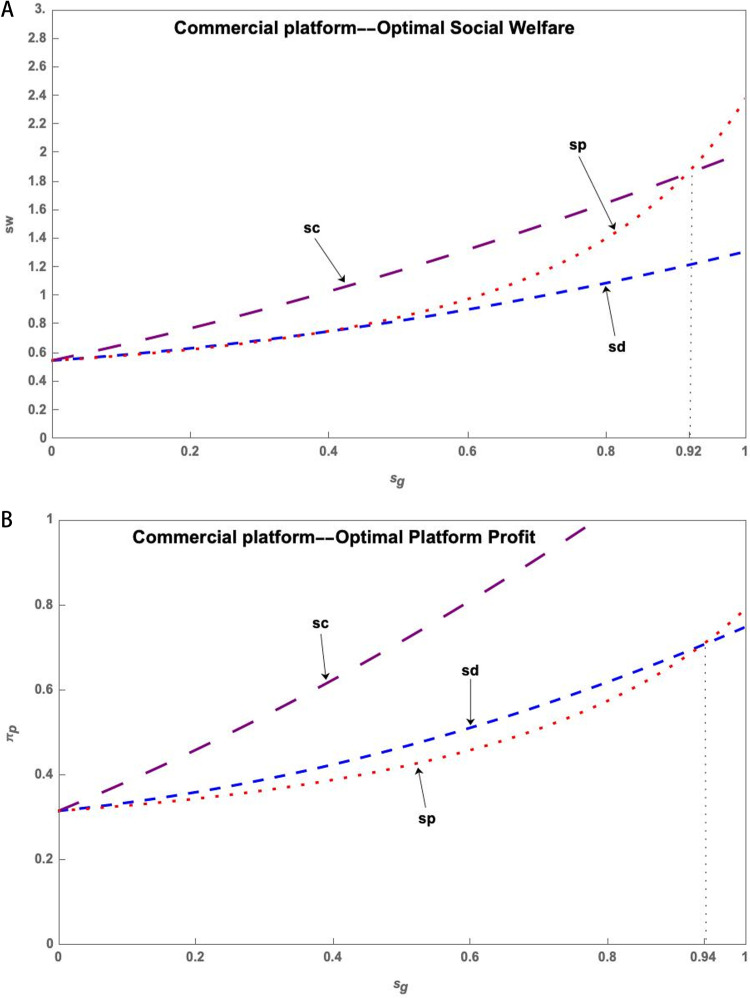
(a) The impact of the $s_{g}$ on ${sw}^{*}$ of Commercial Platform. (b) The impact of the $s_{g}$ on $\pi_{p}^{*}$ of Commercial Platform.

Fig 8 demonstrates that government subsidies to research users in the Commercial Platform can result in the highest social welfare and platform profits.

**Conclusion 10**. Government subsidies to research users can lead to high social welfare and platform profits under the Commercial Platform, and both increase with the amount of government subsidies.

### 6.3 Optimal subsidy strategy

**Fig 9:**
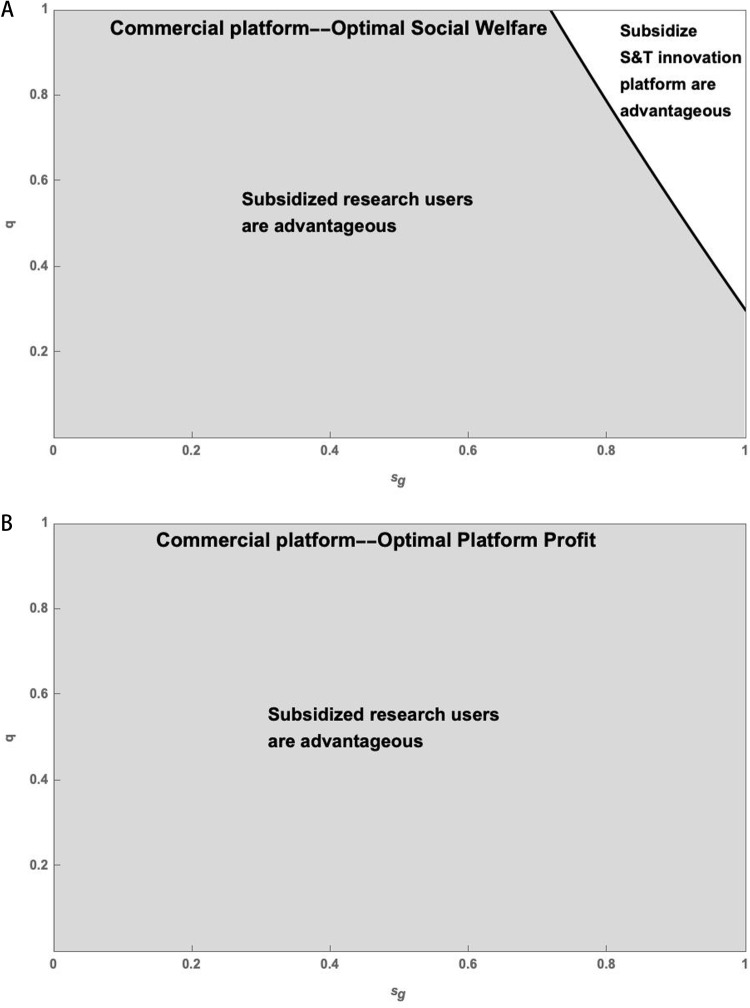
(a) Comparison of the strategies of government subsidy for research users and S&T innovation platform of Commercial Platform--Optimal Social Welfare. (b) Comparison of the strategies of government subsidy for research users and S&T innovation platform of Commercial Platform--Optimal Platform Profit.

Based on the analysis of S&T innovation platform service quality level and government subsidy strength, it is evident that these factors have an impact on the optimal social welfare and platform profit. In the Commercial Platform, government subsidies for research users generally result in the highest social welfare. However, when the subsidy strength is very large, government subsidies for the S&T innovation platform result in the highest social welfare. Thus, simulation is used to compare the predominance of subsidized scientific research users and the subsidized S&T innovation platform, as depicted in Fig 9.

As illustrated in Fig 9, users of government-subsidized research under the Commercial Platform can achieve the highest social welfare and platform profits. However, the maximum social welfare of a government-subsidized S&T innovation platform can only be attained with very high subsidies and service levels.

**Conclusion 11**. To achieve better results, it is recommended that S&T innovation platform operate in Commercial Platform model. Additionally, the government should provide subsidies to research users.

## 7 Conclusion

This study compares and analyzes the pricing strategies of Public Welfare Platforms, Social Enterprise Platforms, and Commercial Platforms. Compared with the existing research results, this paper used optimization theory and game ideas to decide the pricing strategy of S&T platforms, and combined the three operation models of S&T platforms to analyze the most effective government subsidy strategy. It further discusses government subsidies for the three types of innovation platforms to provide theoretical reference for the pricing strategies of S&T innovation platform and the government’s choice of subsidizing entities. This paper innovatively presents the pricing strategy for S&T innovation platforms and compares and analyzes the effect of government subsidies on S&T innovation entities. This paper not only enriches the theoretical research in operation and management but also provides theoretical references for S&T innovation activities. The analysis draws the following conclusions:

(1) In the context of the Public Welfare Platform, optimal equilibrium pricing is observed when the cross-side network effect of platform users is not significant. In order to achieve the goal of maximizing social welfare, the S&T innovation platform must provide commission rebates to resource providers and price subsidies to scientific research users simultaneously. However, this approach may have a significant impact on the platform’s profit. The platform’s profit is a factor that must be considered. The Social Enterprise Platform considers both the social welfare and the profit of the platform enterprise. This model can improve the profit of the S&T innovation platform to a certain extent. The Commercial Platform has the highest platform profit. The platform profit increases with the increase of the platform service level. The Commercial Platform is the most profitable, with the platform profit increasing as the platform service level increases.
(2) When the government subsidizes innovative entities of all three types, the optimal social welfare and platform profits remain consistent with those without subsidies across all three platform operation models.
(3) The Public Welfare Platform indicates that while a small subsidy to the S&T innovation platform may result in greater social welfare, the profit of the S&T innovation platform is lower at this time. Furthermore, when the government subsidizes the three types of innovation subjects, the profit of the platform decreases with the increase of the subsidy.
(4) The Social Enterprise Platform allows for the potential increase in the profit of the S&T innovation platform through government subsidies to resource providers and research users. As the subsidies increase, the government subsidies to resource providers can also result in a positive increase in the platform’s profit. However, this comes at the cost of reduced social welfare.
(5) Under the Commercial Platform, the government subsidizes three types of innovation subjects. As the subsidy strength increases, both social welfare and S&T innovation platform profits increase. Additionally, the government subsidizes research users with the best effect.

The theoretical contributions of this study are mainly: (1) Combining the characteristics of the public welfare platform and the commercial platform, and considering the different operation models of the platforms, this study enriches the pricing strategy of the platforms under the government subsidy scenario through modeling. (2) The service supply chain of S&T innovation platform is subdivided into operation models, and the pricing model of the platforms is portrayed for the first time under different operation models. This improves the theoretical system of the service supply chain of S&T innovation platform. (3) A detailed analysis of different government subsidy strategies is conducted, further validating the government subsidy strategy of the platforms.

Some managerial insights can be gained from this study. Regarding the development cycle of S&T platforms, platforms should adopt different operational models at various stages of development. The conclusions of this paper can provide detailed suggestions for S&T platforms and governments: (1) At the early stage of the development of S&T platforms, to promote the convergence of S&T resources, the platform should adopt a public welfare operating model, and attract more resource providers and research users to join the platform by providing price subsidies to resource providers and research users. At this time, government subsidies to the three types of innovation subjects are not conducive to the growth of profits of S&T platforms. (2) In the middle stage of the development of the S&T platform, the platform should adopt the social enterprise operation model and reduce the commission rebates paid to resource providers, but at this time, government subsidies to the three types of innovative entities still cannot simultaneously balance social welfare and platform profits. (3) In the late stage of the development of the S&T platform, the platform should adopt a commercial mode of operation and increase the membership fee for research users, at which time government subsidies for the three types of innovation subjects can simultaneously enhance social welfare and the profit of the S&T platform, and government subsidies for research users are the most effective.

It can be seen that in the early and middle stages of the development of S&T platforms, price subsidies should be used by the platforms to attract more resource providers and research users to join the platforms, and in the later stages of the development of S&T platforms, the government subsidizes the research users to further enhance the innovation dynamics.

In addition, the study will be able to provide appropriate policy recommendations for regions at different stages of economic development. High-income areas should adopt the Commercial Platform operating model, where the government should subsidize research users; low-income areas should adopt the Public Welfare Platform operating model, where the government should subsidize the S&T innovation platform.

This study concludes that government subsidies to research users are the most effective. Furthermore, the riskiness of science and technology innovation is an important factor of concern for research users. Consequently, the next step in this research is to determine the optimal subsidy strategy for the government to implement for research users, considering the varying levels of risk associated with different types of science and technology innovation activities. In addition to considering the risks of innovation, further consideration needs to be given to how government subsidies can be gradually weakened so that a virtuous cycle in the supply chain of S&T platforms can be realized through a revenue-sharing contract among innovation agents.

## Supporting information

S1 Appendix Proof of the main conclusion. (DOCX)
