# Supplementary material for: Analysis of government subsidy strategies in the supply chain of science and technology innovation platform
Source: PLoS One. 2025 May 16;20(5):e0323627. doi: 10.1371/journal.pone.0323627 (PMC12084066; doi:10.1371/journal.pone.0323627)
Supplement: S1 Appendix — (DOCX) [file pone.0323627.s001.docx]

Appendix

## A.1 Proof of Proposition 1

Substituting Eq. (1) and Eq. (2) into Eq. (3) yields,$\frac{\partial^{2}{SW}^{a}}{\partial p^{2}}=\frac{\alpha_{d}\left( 2\alpha_{c}+\alpha_{d} \right)-1}{{(1-\alpha_{c}\alpha_{d})}^{2}}$, $\left| \begin{matrix} \frac{\partial^{2}{SW}^{a}}{\partial p^{2}} & \frac{\partial^{2}{SW}^{a}}{\partial p\partial w} \\ \frac{\partial^{2}{SW}^{a}}{\partial w\partial p} & \frac{\partial^{2}{SW}^{a}}{\partial w^{2}} \end{matrix} \right|=\frac{1-\left( \alpha_{c}+\alpha_{d} \right)^{2}}{{(1-\alpha_{c}\alpha_{d})}^{2}}$, and thus when $0<\alpha_{c}<1-\alpha_{d}$, $\frac{\partial^{2}{SW}^{a}}{\partial p^{2}}<0$,$\left| \begin{matrix} \frac{\partial^{2}{SW}^{a}}{\partial p^{2}} & \frac{\partial^{2}{SW}^{a}}{\partial p\partial w} \\ \frac{\partial^{2}{SW}^{a}}{\partial w\partial p} & \frac{\partial^{2}{SW}^{a}}{\partial w^{2}} \end{matrix} \right|>0$, the second-order Hessian matrices of $p$, and $w$ with respect to ${SW}^{a}$ are negatively definite, and the joint $\frac{\partial{SW}^{a}}{\partial p}=0$ and $\frac{\partial{SW}^{a}}{\partial w}=0$ yields $p^{a^{*}}$ and $w^{a^{*}}$, and thus there exists a unique equilibrium solution of $p^{a^{*}}$ and $w^{a^{*}}$ that maximizes the social welfare under the Public Welfare Platform mode of operation.

The proof is completed.

## A.2 Proof of Proposition 2

Substitute Eq. (1) and Eq. (2) into Eq. (5) for the inverse solution $\frac{\partial^{2}\pi_{p}^{b}}{\partial w^{2}}=-\frac{2}{1-\alpha_{c}\alpha_{d}}<0$, so there exists a unique equilibrium solution $w^{b^{*}}$ that maximizes $\pi_{p}^{b}$, and according to the FOC condition, making $\frac{\partial\pi_{p}^{b}}{\partial w}=0$ gives $w^{b^{*}}=\frac{p\alpha_{c}-\alpha_{d}(1-p+q-\eta\mu)}{2}$, and substituting it into ${SW}^{b}$ calculates $\frac{\partial^{2}{SW}^{b}}{\partial p^{2}}=\frac{2\left( \alpha_{c}\alpha_{d}\left( 3-\alpha_{d}^{2} \right)-2 \right)-\left( 1-\alpha_{c}^{2} \right)\left( \alpha_{c}^{2}-3\alpha_{d}^{2} \right)}{4{(1-\alpha_{c}\alpha_{d})}^{2}}$, and when $\alpha_{c}\alpha_{d}<\frac{4+\left( 1-\alpha_{c}^{2} \right)\left( \alpha_{c}^{2}-3\alpha_{d}^{2} \right)}{2\left( 3-\alpha_{d}^{2} \right)}$, $\frac{\partial^{2}{SW}^{b}}{\partial p^{2}}<0$ there exists a unique equilibrium solution $p^{b^{*}}$ that maximizes ${SW}^{b}$, and according to the FOC condition, making $\frac{\partial{SW}^{b}}{\partial p}=0$ gives $p^{b^{*}}$, and therefore there exists a unique equilibrium solutions $p^{b^{*}}$ and $w^{b^{*}}$ that maximize social welfare while taking into account the profit of the S&T innovation platform.

The proof is completed.

## A.3 Proof of Proposition 3

Substituting Eq. (1) and Eq. (2) into Eq. (7) yields $\frac{\partial^{2}\pi_{p}^{c}}{\partial p^{2}}=-\frac{2}{1-\alpha_{c}\alpha_{d}}<0$, $\left| \begin{matrix} \frac{\partial^{2}\pi_{p}^{c}}{\partial p^{2}} & \frac{\partial^{2}\pi_{p}^{c}}{\partial p\partial w} \\ \frac{\partial^{2}\pi_{p}^{c}}{\partial w\partial p} & \frac{\partial^{2}\pi_{p}^{c}}{\partial w^{2}} \end{matrix} \right|=\frac{4-{(\alpha_{c}+\alpha_{d})}^{2}}{{(1-\alpha_{c}\alpha_{d})}^{2}}>0$, and thus $\pi_{p}^{c}$ is negative definite with respect to the second-order Hessian matrices of $p$ and $w$, and associating $\frac{\partial\pi_{p}^{c}}{\partial p}=0$ and $\frac{\partial\pi_{p}^{c}}{\partial w}=0$ yields $p^{c^{*}}$ and $w^{c^{*}}$, and thus there exists a unique equilibrium solution of $p^{c^{*}}$ and $w^{c^{*}}$ that maximizes the Social Welfare under the Public Welfare Platform.

The proof is completed.

## A.4 Proof of Property 1

(1) Public Welfare Platform: $\frac{\partial p^{a^{*}}}{\partial q}=-\frac{\alpha_{d}(\alpha_{c}+\alpha_{d})}{1-{(\alpha_{c}+\alpha_{d})}^{2}}<0$, $\frac{\partial w^{a^{*}}}{\partial q}=\frac{\alpha_{c}}{1-{(\alpha_{c}+\alpha_{d})}^{2}}>0$.

(2) Social Enterprise Platform:$\frac{\partial p^{b^{*}}}{\partial q}=\frac{\left( \alpha_{c}+\alpha_{d} \right)\left( \left( \alpha_{c}-2\alpha_{d} \right)\left( \alpha_{c}\alpha_{d}-2 \right)-\alpha_{d} \right)}{2\left( \alpha_{c}\alpha_{d}\left( 3-\alpha_{d}^{2} \right)-2 \right)-\left( 1-\alpha_{c}^{2} \right)\left( \alpha_{c}^{2}-3\alpha_{d}^{2} \right)}$, if $\alpha_{c}>2\alpha_{d}$,$\frac{\partial p^{b^{*}}}{\partial q}>0$, $\frac{\partial w^{b^{*}}}{\partial q}=\frac{2\alpha_{d}-\alpha_{c}\left( \alpha_{c}^{2}+\alpha_{d}^{2} \right)}{2\left( \alpha_{c}\alpha_{d}\left( 3-\alpha_{d}^{2} \right)-2 \right)-\left( 1-\alpha_{c}^{2} \right)\left( \alpha_{c}^{2}-3\alpha_{d}^{2} \right)}$, if $\alpha_{d}>\alpha_{c}$,$\frac{\partial w^{b^{*}}}{\partial q}<0$.

(3) Commercial Platform operating model: $\frac{\partial p^{c^{*}}}{\partial q}=\frac{2-\alpha_{d}(\alpha_{c}+\alpha_{d})}{4-{(\alpha_{c}+\alpha_{d})}^{2}}>0$, $\frac{\partial w^{c^{*}}}{\partial q}=\frac{\alpha_{c}-\alpha_{d}}{4-{(\alpha_{c}+\alpha_{d})}^{2}}$, when $\alpha_{d}<\alpha_{c}$,$\frac{\partial w^{c^{*}}}{\partial q}>0$; when $\alpha_{d}>\alpha_{c}$, $\frac{\partial w^{c^{*}}}{\partial q}<0$.

The proof is completed.

## A.5 Proof of Proposition 4

Substituting Eq. (9) and Eq. (10) into Eq. (13) yields, $\frac{\partial^{2}{SW}^{sda}}{\partial p^{2}}=\frac{\alpha_{d}\left( 2\alpha_{c}+\alpha_{d} \right)-1}{{(1-\alpha_{c}\alpha_{d})}^{2}}$,$\left| \begin{matrix} \frac{\partial^{2}{SW}^{sda}}{\partial p^{2}} & \frac{\partial^{2}{SW}^{sda}}{\partial p\partial w} \\ \frac{\partial^{2}{SW}^{sda}}{\partial w\partial p} & \frac{\partial^{2}{SW}^{sda}}{\partial w^{2}} \end{matrix} \right|=\frac{1-\left( \alpha_{c}+\alpha_{d} \right)^{2}}{{(1-\alpha_{c}\alpha_{d})}^{2}}$, and thus when $0<\alpha_{c}<1-\alpha_{d}$,$\frac{\partial^{2}{SW}^{sda}}{\partial p^{2}}<0$, $\left| \begin{matrix} \frac{\partial^{2}{SW}^{sda}}{\partial p^{2}} & \frac{\partial^{2}{SW}^{sda}}{\partial p\partial w} \\ \frac{\partial^{2}{SW}^{sda}}{\partial w\partial p} & \frac{\partial^{2}{SW}^{sda}}{\partial w^{2}} \end{matrix} \right|>0$, ${SW}^{sda}$the second-order Hessian matrix about $p$ and $w$ is negative definite, and associating $\frac{\partial{SW}^{sda}}{\partial p}=0$ and $\frac{\partial{SW}^{sda}}{\partial w}=0$ ,yields $p^{{sda}^{*}}$ and $w^{{sda}^{*}}$, and thus there exists the unique equilibrium solutions,$p^{{sda}^{*}}$ and $w^{{sda}^{*}}$, which make the subsidized resource providers under the Public Welfare Platform mode of operation to achieve the social welfare Max.

The proof is completed.

## A.6 Proof of Proposition 5

Substituting Eq. (11) and Eq. (12) into Eq. (15) yields, $\frac{\partial^{2}{SW}^{sca}}{\partial p^{2}}=\frac{\alpha_{d}\left( 2\alpha_{c}+\alpha_{d} \right)-1}{{(1-\alpha_{c}\alpha_{d})}^{2}}$, $\left| \begin{matrix} \frac{\partial^{2}{SW}^{sca}}{\partial p^{2}} & \frac{\partial^{2}{SW}^{sca}}{\partial p\partial w} \\ \frac{\partial^{2}{SW}^{sca}}{\partial w\partial p} & \frac{\partial^{2}{SW}^{sca}}{\partial w^{2}} \end{matrix} \right|=\frac{1-\left( \alpha_{c}+\alpha_{d} \right)^{2}}{{(1-\alpha_{c}\alpha_{d})}^{2}}$, and thus when $0<\alpha_{c}<1-\alpha_{d}$,$\frac{\partial^{2}{SW}^{sca}}{\partial p^{2}}<0$, $\left| \begin{matrix} \frac{\partial^{2}{SW}^{sca}}{\partial p^{2}} & \frac{\partial^{2}{SW}^{sca}}{\partial p\partial w} \\ \frac{\partial^{2}{SW}^{sca}}{\partial w\partial p} & \frac{\partial^{2}{SW}^{sca}}{\partial w^{2}} \end{matrix} \right|>0$, the second-order Hessian matrices of $p$, and $w$ with respect to ${SW}^{sca}$ are negatively definite, and the joint $\frac{\partial{SW}^{sca}}{\partial p}=0$ and $\frac{\partial{SW}^{sca}}{\partial w}=0$ yields $p^{{sca}^{*}}$ and $w^{{sca}^{*}}$, and thus there exists a unique equilibrium solution of $p^{{sca}^{*}}$ and $w^{{sca}^{*}}$ making the government subsidize scientific research users to achieve the maximum social welfare under the Public Welfare Platform mode of operation.

The proof is completed.

## A.7 Proof of Proposition 6

Substituting Eq. (1) and Eq. (2) into Eq. (17) yields, $\frac{\partial^{2}{SW}^{spa}}{\partial p^{2}}=\frac{\alpha_{d}\left( 2\left( \alpha_{c}+s_{g} \right)+\alpha_{d} \right)-1}{{(1-\alpha_{c}\alpha_{d})}^{2}}$, $\left| \begin{matrix} \frac{\partial^{2}{SW}^{spa}}{\partial p^{2}} & \frac{\partial^{2}{SW}^{spa}}{\partial p\partial w} \\ \frac{\partial^{2}{SW}^{spa}}{\partial w\partial p} & \frac{\partial^{2}{SW}^{spa}}{\partial w^{2}} \end{matrix} \right|=\frac{1-\left( \alpha_{c}+\alpha_{d}+s_{g} \right)^{2}}{{(1-\alpha_{c}\alpha_{d})}^{2}}$, and thus when$0<\alpha_{c}<1-\alpha_{d}-s_{g}$,$\frac{\partial^{2}{SW}^{spa}}{\partial p^{2}}<0$,$\left| \begin{matrix} \frac{\partial^{2}{SW}^{spa}}{\partial p^{2}} & \frac{\partial^{2}{SW}^{spa}}{\partial p\partial w} \\ \frac{\partial^{2}{SW}^{spa}}{\partial w\partial p} & \frac{\partial^{2}{SW}^{spa}}{\partial w^{2}} \end{matrix} \right|>0$, the second-order Hessian matrices of $p$, and $w$ with respect to ${SW}^{spa}$ are negatively definite, and the joint $\frac{\partial{SW}^{spa}}{\partial p}=0$ and $\frac{\partial{SW}^{spa}}{\partial w}=0$ yields $p^{{spa}^{*}}$ and $w^{{spa}^{*}}$, and thus there exists a unique equilibrium solution of $p^{{spa}^{*}}$ and $w^{{spa}^{*}}$ making the government subsidize S&T innovation platform to achieve the maximum social welfare under the Public Welfare Platform mode of operation.

The proof is completed.

## A.8 Proof of Property 2

(1) Government subsidizes resource providers:$\frac{\partial p^{{sda}^{*}}}{\partial q}=-\frac{\alpha_{d}(\alpha_{c}+\alpha_{d})}{1-{(\alpha_{c}+\alpha_{d})}^{2}}<0$, $\frac{\partial w^{sda^{*}}}{\partial q}=\frac{\alpha_{c}}{1-{(\alpha_{c}+\alpha_{d})}^{2}}>0$; $\frac{\partial p^{{sda}^{*}}}{\partial s_{g}}=-\frac{\alpha_{d}}{1-{(\alpha_{c}+\alpha_{d})}^{2}}<0$, $\frac{\partial w^{sda^{*}}}{\partial s_{g}}=\frac{\alpha_{c}(\alpha_{c}+\alpha_{d})}{1-{(\alpha_{c}+\alpha_{d})}^{2}}>0$.

(2) Government Subsidized Research Users:$\frac{\partial p^{{sca}^{*}}}{\partial q}=-\frac{\alpha_{d}(\alpha_{c}+\alpha_{d})}{1-{(\alpha_{c}+\alpha_{d})}^{2}}<0$, $\frac{\partial w^{sca^{*}}}{\partial q}=\frac{\alpha_{c}}{1-{(\alpha_{c}+\alpha_{d})}^{2}}>0$; $\frac{\partial p^{{sca}^{*}}}{\partial s_{g}}=-\frac{\alpha_{d}(\alpha_{c}+\alpha_{d})}{1-{(\alpha_{c}+\alpha_{d})}^{2}}<0$, $\frac{\partial w^{sca^{*}}}{\partial s_{g}}=\frac{\alpha_{c}}{1-{(\alpha_{c}+\alpha_{d})}^{2}}>0$.

(3) Government subsidized S&T innovation platform:$\frac{\partial p^{{spa}^{*}}}{\partial q}=-\frac{(s_{g}+\alpha_{d})(s_{g}+\alpha_{c}+\alpha_{d})}{1-{(s_{g}+\alpha_{c}+\alpha_{d})}^{2}}<0$, $\frac{\partial w^{spa^{*}}}{\partial q}=\frac{s_{g}+\alpha_{c}}{1-{(s_{g}+\alpha_{c}+\alpha_{d})}^{2}}>0, \frac{\partial p^{{spa}^{*}}}{\partial s_{g}}=\frac{\left( 1+q-\eta\mu\right)\left( \alpha_{c}+\left( s_{g}+\alpha_{c}+\alpha_{d} \right)\left( \alpha_{c}\left( s_{g}+\alpha_{c}+\alpha_{d} \right)-2 \right) \right)}{\left( 1-{(s_{g}+\alpha_{c}+\alpha_{d})}^{2} \right)^{2}}<0$, $\frac{\partial w^{spa^{*}}}{\partial s_{g}}=\frac{\left( 1+q-\eta\mu\right)\left( 1-\alpha_{d}^{2}+\left( s_{g}+\alpha_{c} \right)^{2} \right)}{\left( 1-\left( s_{g}+\alpha_{c}+\alpha_{d} \right)^{2} \right)^{2}}>0$.

The proof is completed.

## A.9 Proof of Proposition 7

Substitute Eq. (9) and Eq. (10) into Eq. (19) for the inverse solution $\frac{\partial^{2}\pi_{p}^{sdb}}{\partial w^{2}}=-\frac{2}{1-\alpha_{c}\alpha_{d}}<0$, so there exists a unique equilibrium solution $w^{{sdb}^{*}}$ that maximizes $\pi_{p}^{sdb}$, and according to the FOC condition, making $\frac{\partial\pi_{p}^{sdb}}{\partial w}=0$ gives $w^{{sdb}^{*}}=\frac{p\alpha_{c}-\alpha_{d}(1-p+q-\eta\mu)-s_{g}}{2}$, and substituting it into ${SW}^{sdb}$ calculates $\frac{\partial^{2}S^{sdb}}{\partial p^{2}}=\frac{2\left( \alpha_{c}\alpha_{d}\left( 3-\alpha_{d}^{2} \right)-2 \right)-\left( 1-\alpha_{c}^{2} \right)\left( \alpha_{c}^{2}-3\alpha_{d}^{2} \right)}{4{(1-\alpha_{c}\alpha_{d})}^{2}}$, and when $\alpha_{c}\alpha_{d}<\frac{4+\left( 1-\alpha_{c}^{2} \right)\left( \alpha_{c}^{2}-3\alpha_{d}^{2} \right)}{2\left( 3-\alpha_{d}^{2} \right)}$, there exists a unique equilibrium solution $p^{{sdb}^{*}}$ that maximizes ${SW}^{sdb}$, and according to the FOC condition, making $\frac{\partial{SW}^{sdb}}{\partial p}=0$ gives $p^{{sdb}^{*}}$, and therefore there exists a unique equilibrium solutions $p^{{sdb}^{*}}$ and $w^{{sdb}^{*}}$ that maximize social welfare while taking into account the profit of the S&T innovation platform.

The proof is completed.

## A.10 Proof of Proposition 8

Substitute Eq. (11) and Eq. (12) into Eq. (21) for the inverse solution $\frac{\partial^{2}\pi_{p}^{scb}}{\partial w^{2}}=-\frac{2}{1-\alpha_{c}\alpha_{d}}<0$, so there exists a unique equilibrium solution $w^{{scb}^{*}}$ that maximizes $\pi_{p}^{scb}$, and according to the FOC condition, making $\frac{\partial\pi_{p}^{scb}}{\partial w}=0$ gives $w^{{scb}^{*}}=\frac{p\alpha_{c}-\alpha_{d}(1-p+q-\eta\mu+s_{g})}{2}$, and substituting it into ${SW}^{scb}$ calculates $\frac{\partial^{2}{SW}^{scb}}{\partial p^{2}}=\frac{2\left( \alpha_{c}\alpha_{d}\left( 3-\alpha_{d}^{2} \right)-2 \right)-\left( 1-\alpha_{c}^{2} \right)\left( \alpha_{c}^{2}-3\alpha_{d}^{2} \right)}{4{(1-\alpha_{c}\alpha_{d})}^{2}}$, and when $\alpha_{c}\alpha_{d}<\frac{4+\left( 1-\alpha_{c}^{2} \right)\left( \alpha_{c}^{2}-3\alpha_{d}^{2} \right)}{2\left( 3-\alpha_{d}^{2} \right)}$, there exists a unique equilibrium solution $p^{{scb}^{*}}$ that maximizes ${SW}^{scb}$, and according to the FOC condition, making $\frac{\partial{SW}^{scb}}{\partial p}=0$ gives $p^{{scb}^{*}}$, and therefore there exists a unique equilibrium solutions $p^{{scb}^{*}}$ and $w^{{scb}^{*}}$ that maximize social welfare while taking into account the profit of the S&T innovation platform.

The proof is completed.

## A.11 Proof of Proposition 9

Substitute Eq. (1) and Eq. (2) into Eq. (23) for the inverse solution $\frac{\partial^{2}\pi_{p}^{spb}}{\partial w^{2}}=-\frac{2\left( 1-\alpha_{c}\left( s_{g}+\alpha_{d} \right) \right)}{\left( 1-\alpha_{c}\alpha_{d} \right)^{2}}<0$, so there exists a unique equilibrium solution $w^{{spb}^{*}}$ that maximizes $\pi_{p}^{spb}$, and according to the FOC condition, making $\frac{\partial\pi_{p}^{spb}}{\partial w}=0$ gives $w^{{spb}^{*}}=\frac{\left( p\alpha_{c}-\alpha_{d}\left( 1-p+q-\eta\mu\right) \right)\left( 1-\alpha_{c}\alpha_{d} \right)+s_{g}\left( 1-p+q-\eta\mu\right)\left( 1+\alpha_{c}\alpha_{d} \right)}{2\left( 1-\alpha_{c}\left( s_{g}+\alpha_{d} \right) \right)}$, and substituting it into ${SW}^{spb}$ calculates $\frac{\partial^{2}{SW}^{spb}}{\partial p^{2}}=\frac{\left( 1+\alpha_{c}^{2} \right)\left( 1-\alpha_{c}^{2} \right)^{2}-\left( 1+2s_{g}\alpha_{c}+3\alpha_{c}^{2}+2\alpha_{c}\alpha_{d} \right)\left( 1-\alpha_{c}\left( s_{g}+\alpha_{d} \right) \right)^{2}}{4\alpha_{c}^{2}\left( 1-\alpha_{c}\left( s_{g}+\alpha_{d} \right) \right)^{2}}$, and when $\left( 1-\alpha_{c}^{2} \right)^{2}\left( 1+\alpha_{c}^{2} \right)<\left( 1+2s_{g}\alpha_{c}+3\alpha_{c}^{2}+2\alpha_{c}\alpha_{d} \right)\left( 1-\alpha_{c}\left( s_{g}+\alpha_{d} \right) \right)^{2}$, there exists a unique equilibrium solution $p^{{spb}^{*}}$ that maximizes ${SW}^{spb}$, and according to the FOC condition, making $\frac{\partial{SW}^{spb}}{\partial p}=0$ gives $p^{{spb}^{*}}$, and therefore there exists a unique equilibrium solutions $p^{{spb}^{*}}$ and $w^{{spb}^{*}}$ that maximize social welfare while taking into account the profit of the S&T innovation platform.

The proof is completed.

## A.12 Proof of Property 3

(1) Government Subsidized Resource Providers: $\frac{\partial p^{{sdb}^{*}}}{\partial q}=\frac{\left( \alpha_{c}+\alpha_{d} \right)\left( \left( \alpha_{c}+\alpha_{d} \right)\left( \left( \alpha_{c}-2\alpha_{d} \right)\left( \alpha_{c}\alpha_{d}-2 \right)-\alpha_{d} \right) \right)}{2\left( \alpha_{c}\alpha_{d}\left( 3-\alpha_{d}^{2} \right)-2 \right)-\left( 1-\alpha_{c}^{2} \right)\left( \alpha_{c}^{2}-3\alpha_{d}^{2} \right)}$, $\frac{\partial w^{{sdb}^{*}}}{\partial q}=\frac{2\alpha_{d}-\alpha_{c}\left( \alpha_{c}^{2}+\alpha_{d}^{2} \right)}{2\left( \alpha_{c}\alpha_{d}\left( 3-\alpha_{d}^{2} \right)-2 \right)-\left( 1-\alpha_{c}^{2} \right)\left( \alpha_{c}^{2}-3\alpha_{d}^{2} \right)}$, $\frac{\partial p^{{sdb}^{*}}}{\partial s_{g}}=\frac{\alpha_{d}\left( 3+\alpha_{c}^{2} \right)-\alpha_{c}\left( 2\alpha_{d}^{2}+\alpha_{c}^{2}+1 \right)}{2\left( \alpha_{c}\alpha_{d}\left( 3-\alpha_{d}^{2} \right)-2 \right)-\left( 1-\alpha_{c}^{2} \right)\left( \alpha_{c}^{2}-3\alpha_{d}^{2} \right)}$, $\frac{\partial w^{{sdb}^{*}}}{\partial s_{g}}=\frac{\left( 1-\alpha_{c}\alpha_{d} \right)^{2}+1-\alpha_{c}^{4}}{2\left( \alpha_{c}\alpha_{d}\left( 3-\alpha_{d}^{2} \right)-2 \right)-\left( 1-\alpha_{c}^{2} \right)\left( \alpha_{c}^{2}-3\alpha_{d}^{2} \right)}<0$, if $\alpha_{c}>2\alpha_{d}$,$\frac{\partial p^{{sdb}^{*}}}{\partial q}>0$; if$\alpha_{d}>\alpha_{c}$,$\frac{\partial w^{{sdb}^{*}}}{\partial q}<0$, $\frac{\partial p^{{sdb}^{*}}}{\partial s_{g}}<0$.

(2) Users of government-subsidized research: $\frac{\partial p^{{scb}^{*}}}{\partial q}=\frac{\left( \alpha_{c}+\alpha_{d} \right)\left( \left( \alpha_{c}-2\alpha_{d} \right)\left( \alpha_{c}\alpha_{d}-2 \right)-\alpha_{d} \right)}{2\left( \alpha_{c}\alpha_{d}\left( 3-\alpha_{d}^{2} \right)-2 \right)-\left( 1-\alpha_{c}^{2} \right)\left( \alpha_{c}^{2}-3\alpha_{d}^{2} \right)}$, $\frac{\partial w^{{scb}^{*}}}{\partial q}=\frac{2\alpha_{d}-\alpha_{c}\left( \alpha_{c}^{2}+\alpha_{d}^{2} \right)}{2\left( \alpha_{c}\alpha_{d}\left( 3-\alpha_{d}^{2} \right)-2 \right)-\left( 1-\alpha_{c}^{2} \right)\left( \alpha_{c}^{2}-3\alpha_{d}^{2} \right)}, \frac{\partial p^{{scb}^{*}}}{\partial s_{g}}=\frac{\left( \alpha_{c}+\alpha_{d} \right)\left( \left( \alpha_{c}-2\alpha_{d} \right)\left( \alpha_{c}\alpha_{d}-2 \right)-\alpha_{d} \right)}{2\left( \alpha_{c}\alpha_{d}\left( 3-\alpha_{d}^{2} \right)-2 \right)-\left( 1-\alpha_{c}^{2} \right)\left( \alpha_{c}^{2}-3\alpha_{d}^{2} \right)}$, $\frac{\partial w^{{scb}^{*}}}{\partial s_{g}}=\frac{2\alpha_{d}-\alpha_{c}\left( \alpha_{c}^{2}+\alpha_{d}^{2} \right)}{2\left( \alpha_{c}\alpha_{d}\left( 3-\alpha_{d}^{2} \right)-2 \right)-\left( 1-\alpha_{c}^{2} \right)\left( \alpha_{c}^{2}-3\alpha_{d}^{2} \right)}$, if $\alpha_{c}>2\alpha_{d},\frac{\partial p^{{scb}^{*}}}{\partial q}>0$, $\frac{\partial p^{{scb}^{*}}}{\partial s_{g}}>0$; if $\alpha_{d}>\alpha_{c}$, $\frac{\partial w^{{scb}^{*}}}{\partial q}<0$, $\frac{\partial w^{{scb}^{*}}}{\partial s_{g}}<0$.

The proof is completed.

## A.13 Proof of Proposition 10

Substituting Eq. (9) and Eq. (10) into Eq. (25) yields $\frac{\partial^{2}\pi_{p}^{sdc}}{\partial p^{2}}=-\frac{2}{1-\alpha_{c}\alpha_{d}}<0$, $\left| \begin{matrix} \frac{\partial^{2}\pi_{p}^{sdc}}{\partial p^{2}} & \frac{\partial^{2}\pi_{p}^{sdc}}{\partial p\partial w} \\ \frac{\partial^{2}\pi_{p}^{sdc}}{\partial w\partial p} & \frac{\partial^{2}\pi_{p}^{sdc}}{\partial w^{2}} \end{matrix} \right|=\frac{4-{(\alpha_{c}+\alpha_{d})}^{2}}{{(1-\alpha_{c}\alpha_{d})}^{2}}>0$, and thus $\pi_{p}^{sdc}$ is negative definite with respect to the second-order Hessian matrices of $p$ and $w$, and associating $\frac{\partial\pi_{p}^{sdc}}{\partial p}=0$ and $\frac{\partial\pi_{p}^{sdc}}{\partial w}=0$ yields $p^{{sdc}^{*}}$ and $w^{{sdc}^{*}}$, and thus there exists a unique equilibrium solution of $p^{{sdc}^{*}}$ and $w^{{sdc}^{*}}$ makes the Commercial Platform mode of operation maximize the platform profit.

The proof is completed.

## A.14 Proof of Proposition 11

Substituting Eq. (11) and Eq. (12) into Eq. (27) yields $\frac{\partial^{2}\pi_{p}^{scc}}{\partial p^{2}}=-\frac{2}{1-\alpha_{c}\alpha_{d}}<0$, $\left| \begin{matrix} \frac{\partial^{2}\pi_{p}^{scc}}{\partial p^{2}} & \frac{\partial^{2}\pi_{p}^{scc}}{\partial p\partial w} \\ \frac{\partial^{2}\pi_{p}^{scc}}{\partial w\partial p} & \frac{\partial^{2}\pi_{p}^{scc}}{\partial w^{2}} \end{matrix} \right|=\frac{4-{(\alpha_{c}+\alpha_{d})}^{2}}{{(1-\alpha_{c}\alpha_{d})}^{2}}>0$, and thus $\pi_{p}^{scc}$ is negative definite with respect to the second-order Hessian matrices of $p$ and $w$, and associating $\frac{\partial\pi_{p}^{scc}}{\partial p}=0$ and $\frac{\partial\pi_{p}^{scc}}{\partial w}=0$ yields $p^{{scc}^{*}}$ and $w^{{scc}^{*}}$, and thus there exists a unique equilibrium solution of $p^{{scc}^{*}}$ and $w^{{scc}^{*}}$ makes the Public Welfare Platform in which the government subsidizes scientific research users to maximize the platform's profit.

The proof is completed.

## A.15 Proof of Proposition 12

Substituting Eq. (1) and Eq. (2) into Eq. (29) yields $\frac{\partial^{2}\pi_{p}^{spc}}{\partial p^{2}}=-\frac{2-2\alpha_{d}(s_{g}+\alpha_{c})}{\left( 1-\alpha_{c}\alpha_{d} \right)^{2}}<0$, $\left| \begin{matrix} \frac{\partial^{2}\pi_{p}^{spc}}{\partial p^{2}} & \frac{\partial^{2}\pi_{p}^{spc}}{\partial p\partial w} \\ \frac{\partial^{2}\pi_{p}^{spc}}{\partial w\partial p} & \frac{\partial^{2}\pi_{p}^{spc}}{\partial w^{2}} \end{matrix} \right|=\frac{4-{(\alpha_{c}+\alpha_{d})}^{2}}{{(1-\alpha_{c}\alpha_{d})}^{2}}>0$, and thus $\pi_{p}^{spc}$ is negative definite with respect to the second-order Hessian matrices of $p$ and $w$, and associating $\frac{\partial\pi_{p}^{spc}}{\partial p}=0$ and $\frac{\partial\pi_{p}^{spc}}{\partial w}=0$ yields $p^{{spc}^{*}}$ and $w^{{spc}^{*}}$, and thus there exists a unique equilibrium solution of $p^{{spc}^{*}}$ and $w^{{spc}^{*}}$ makes the Public Welfare Platform in which the government subsidizes scientific research users to maximize the platform's profit.

The proof is completed.

## A.16 Proof of Property 4

(1) Government subsidized resource providers:$\frac{\partial p^{{sdc}^{*}}}{\partial q}=\frac{2-\alpha_{d}(\alpha_{c}+\alpha_{d})}{4-{(\alpha_{c}+\alpha_{d})}^{2}}>0$, $\frac{\partial w^{sdc^{*}}}{\partial q}=\frac{\alpha_{c}-\alpha_{d}}{4-{(\alpha_{c}+\alpha_{d})}^{2}}$; $\frac{\partial p^{{sdc}^{*}}}{\partial s_{g}}=\frac{\alpha_{c}-\alpha_{d}}{4-{(\alpha_{c}+\alpha_{d})}^{2}}, \frac{\partial w^{sdc^{*}}}{\partial s_{g}}=-\frac{2-\alpha_{c}\left( \alpha_{c}+\alpha_{d} \right)}{4-\left( \alpha_{c}+\alpha_{d} \right)^{2}}<0.$If$<\alpha_{d}$, $\frac{\partial w^{{sdc}^{*}}}{\partial q}<0, \frac{\partial p^{{sdc}^{*}}}{\partial s_{g}}<0$, if $\alpha_{c}>\alpha_{d}$,$\frac{\partial w^{sdc^{*}}}{\partial q}>0, \frac{\partial p^{{sdc}^{*}}}{\partial s_{g}}>0$.

(2) Government subsidized research users:$\frac{\partial p^{{scc}^{*}}}{\partial q}=\frac{2-\alpha_{d}(\alpha_{c}+\alpha_{d})}{4-{(\alpha_{c}+\alpha_{d})}^{2}}>0, \frac{\partial w^{scc^{*}}}{\partial q}=\frac{\alpha_{c}-\alpha_{d}}{4-{(\alpha_{c}+\alpha_{d})}^{2}}$; $\frac{\partial p^{{scc}^{*}}}{\partial s_{g}}=\frac{2-\alpha_{d}(\alpha_{c}+\alpha_{d})}{4-{(\alpha_{c}+\alpha_{d})}^{2}}>0$, $\frac{\partial w^{scc^{*}}}{\partial s_{g}}=\frac{\alpha_{c}-\alpha_{d}}{4-{(\alpha_{c}+\alpha_{d})}^{2}}$. If $\alpha_{c}<\alpha_{d}$, $\frac{\partial w^{scc^{*}}}{\partial q}<0, \frac{\partial w^{scc^{*}}}{\partial s_{g}}<0$, if $\alpha_{c}>\alpha_{d}$, $\frac{\partial w^{scc^{*}}}{\partial q}>0, \frac{\partial w^{scc^{*}}}{\partial s_{g}}>0$.

(3) Government-subsidized S&T innovation platform:$\frac{\partial p^{{spc}^{*}}}{\partial q}=\frac{2-(s_{g}+\alpha_{d})(s_{g}+\alpha_{c}+\alpha_{d})}{4-{(s_{g}+\alpha_{c}+\alpha_{d})}^{2}}>0$, $\frac{\partial w^{spc^{*}}}{\partial q}=\frac{s_{g}+\alpha_{c}-\alpha_{d}}{4-\left( s_{g}+\alpha_{c}+\alpha_{d} \right)^{2}}$, if $\alpha_{d}<\alpha_{c}+s_{g},\frac{\partial w^{spc^{*}}}{\partial q}>0,$if $\alpha_{d}>\alpha_{c}+s_{g}, \frac{\partial w^{spc^{*}}}{\partial q}<0$; $\frac{\partial p^{{spc}^{*}}}{\partial s_{g}}=\frac{\left( 1+q-\eta\mu\right)\left( \alpha_{c}\left( \alpha_{c}+\alpha_{d}+s_{g} \right)^{2}-4\left( s_{g}+\alpha_{d} \right) \right)}{\left( 4-\left( s_{g}+\alpha_{c}+\alpha_{d} \right)^{2} \right)^{2}}<0$,$\frac{\partial w^{spc^{*}}}{\partial s_{g}}=\frac{\left( 1+q-\eta\mu\right)\left( 4+\left( s_{g}+\alpha_{c}-3\alpha_{d} \right)\left( s_{g}+\alpha_{c}+\alpha_{d} \right) \right)}{\left( 4-\left( s_{g}+\alpha_{c}+\alpha_{d} \right)^{2} \right)^{2}}>0$.

The proof is completed.

## A.17 Proof of Property 5

(1) Compare the optimal membership fee price

$p^{b^{*}}-p^{a^{*}}=-\frac{\left( 1+q-\eta\mu\right)\left( 1-\alpha_{c}^{2} \right)\left( \alpha_{c}+\alpha_{d} \right)\left( 2\alpha_{c}+\alpha_{d} \right)}{\left( 1-\left( \alpha_{c}+\alpha_{d} \right)^{2} \right)\left( 2\left( \alpha_{c}\alpha_{d}\left( 3-\alpha_{d}^{2} \right)-2 \right)-\left( 1-\alpha_{c}^{2} \right)\left( \alpha_{c}^{2}-3\alpha_{d}^{2} \right) \right)}>0$,

$p^{c^{*}}-p^{a^{*}}=\frac{\left( 1+q-\eta\mu\right)\left( 2\left( 1-\alpha_{c}^{2} \right)-\alpha_{d}\left( \alpha_{c}-\alpha_{d} \right) \right)}{\left( 1-\left( \alpha_{c}+\alpha_{d} \right)^{2} \right)\left( 4-\left( \alpha_{c}+\alpha_{d} \right)^{2} \right)}>0$,

$p^{c^{*}}-p^{b^{*}}=-\frac{2\left( 1+q-\eta\mu\right)\left( 1-\alpha_{c}\alpha_{d} \right)\left( 4\left( 1-\alpha_{c}^{2} \right)+\left( \alpha_{c}+\alpha_{d} \right)^{2} \right)}{\left( 4-\left( \alpha_{c}+\alpha_{d} \right)^{2} \right)\left( 2\left( \alpha_{c}\alpha_{d}\left( 3-\alpha_{d}^{2} \right)-2 \right)-\left( 1-\alpha_{c}^{2} \right)\left( \alpha_{c}^{2}-3\alpha_{d}^{2} \right) \right)}>0$,

Therefore $p^{c^{*}}>p^{b^{*}}>p^{a^{*}}$.

(2) Compare the optimal commission rebate

$w^{b^{*}}-w^{a^{*}}=\frac{\left( 1+q-\eta\mu\right)\left( \left( 2\alpha_{c}+\alpha_{d} \right)\left( 2\left( 1+\alpha_{c}^{2} \right)-\left( \alpha_{c}+\alpha_{d} \right)^{2}\left( 2-\alpha_{c}\alpha_{d} \right) \right) \right)}{\left( 1-\left( \alpha_{c}+\alpha_{d} \right)^{2} \right)\left( 2\left( \alpha_{c}\alpha_{d}\left( 3-\alpha_{d}^{2} \right)-2 \right)-\left( 1-\alpha_{c}^{2} \right)\left( \alpha_{c}^{2}-3\alpha_{d}^{2} \right) \right)}<0$,

$w^{c^{*}}-w^{a^{*}}=-\frac{\left( 1+q-\eta\mu\right)\left( \alpha_{c}\left( 1-\alpha_{c}\alpha_{d} \right)+\left( 2\alpha_{c}+\alpha_{d} \right)\left( 1-\alpha_{d}^{2} \right) \right)}{\left( 1-\left( \alpha_{c}+\alpha_{d} \right)^{2} \right)\left( 4-\left( \alpha_{c}+\alpha_{d} \right)^{2} \right)}<0$,

$w^{c^{*}}-w^{b^{*}}=-\frac{\left( \alpha_{c}+\alpha_{d} \right)\left( 1-\alpha_{c}\alpha_{d} \right)\left( 4-\left( 3\alpha_{c}-\alpha_{d} \right)\left( \alpha_{c}+\alpha_{d} \right) \right)}{\left( 4-\left( \alpha_{c}+\alpha_{d} \right)^{2} \right)\left( 2\left( \alpha_{c}\alpha_{d}\left( 3-\alpha_{d}^{2} \right)-2 \right)-\left( 1-\alpha_{c}^{2} \right)\left( \alpha_{c}^{2}-3\alpha_{d}^{2} \right) \right)}>0$,

Therefore $w^{b^{*}}<w^{c^{*}}<w^{a^{*}}$.

The proof is completed.

## A.18 Proof of Property 6

(1) Research user fees. $p^{sda^{*}}-p^{sca^{*}}=\frac{s_{g}\alpha_{d}\left( \alpha_{c}+\alpha_{d}-1 \right)}{1-\left( \alpha_{c}+\alpha_{d} \right)^{2}}<0$, $p^{spa^{*}}-p^{sda^{*}}=\frac{-s_{g}\left( \alpha_{d}\left( q-\eta\mu\right)+\alpha_{d}\left( \alpha_{c}+\alpha_{d}+s_{g} \right)^{2}+\left( 1+q-\eta\mu\right)\left( \alpha_{c}+\alpha_{d}+s_{g} \right)\left( 1-\alpha_{c}\left( \alpha_{c}+\alpha_{d} \right) \right) \right)}{\left( 1-\left( \alpha_{c}+\alpha_{d} \right)^{2} \right)\left( 1-\left( \alpha_{c}+\alpha_{d}+s_{g} \right)^{2} \right)}<0$, $p^{sca^{*}}-p^{a^{*}}=\frac{-s_{g}\alpha_{d}\left( \alpha_{c}+\alpha_{d} \right)}{1-\left( \alpha_{c}+\alpha_{d} \right)^{2}}<0$, therefore $p^{spa^{*}}<p^{sda^{*}}<p^{sca^{*}}<p^{a^{*}}<0$.

(2) Provider commission rebates.$w^{sda^{*}}-w^{a^{*}}=\frac{s_{g}\alpha_{c}\left( \alpha_{c}+\alpha_{d} \right)}{1-\left( \alpha_{c}+\alpha_{d} \right)^{2}}>0$, $w^{sca^{*}}-w^{sda^{*}}=\frac{s_{g}\alpha_{c}\left( 1-\alpha_{c}-\alpha_{d} \right)}{1-\left( \alpha_{c}+\alpha_{d} \right)^{2}}>0, w^{spa^{*}}-w^{sca^{*}}=\frac{s_{g}\left( 1+q-\eta\mu\right)\left( 1-\alpha_{d}^{2}+\alpha_{c}^{2}+s_{g}\alpha_{c} \right)-s_{g}\alpha_{c}\left( 1-\left( \alpha_{c}+\alpha_{d}+s_{g} \right)^{2} \right)}{\left( 1-\left( \alpha_{c}+\alpha_{d}+s_{g} \right)^{2} \right)\left( 1-\left( \alpha_{c}+\alpha_{d} \right)^{2} \right)}>0,$therefore $w^{spa^{*}}>w^{sca^{*}}>w^{sda^{*}}>w^{a^{*}}>0$.

The proof is completed.

## A.19 Proof of Property 7

(1) No government subsidy.$\left| p^{a^{*}} \right|-w^{a^{*}}=\frac{\left( 1+q-\eta\mu\right)\left( \alpha_{d}^{2}-\alpha_{c}\left( 1-\alpha_{d} \right) \right)}{1-\left( \alpha_{c}+\alpha_{d} \right)^{2}}$, $\alpha_{c}<1-\alpha_{d}$ by Proposition 1 , therefore if $\alpha_{d}>\frac{1}{2}$, $\left| p^{a^{*}} \right|-w^{a^{*}}>\frac{\left( 1+q-\eta\mu\right)\left( 2\alpha_{d}-1 \right)}{1-\left( \alpha_{c}+\alpha_{d} \right)^{2}}>0$.

(2) Subsidized resource providers.$\left| p^{sda^{*}} \right|-w^{sda^{*}}=\frac{\left( 1+q-\eta\mu\right)\left( \alpha_{d}^{2}-\alpha_{c}\left( 1-\alpha_{d} \right) \right)+s_{g}\left( \alpha_{d}\left( 1-\alpha_{c} \right)-\alpha_{c}^{2} \right)}{1-\left( \alpha_{c}+\alpha_{d} \right)^{2}}$, $0<\alpha_{c}<1-\alpha_{d}$by Proposition 4, therefore if $\alpha_{d}>\frac{1}{2}$,$\left| p^{sda^{*}} \right|-w^{sda^{*}}>0$.

(3) Subsidizing research users.$\left| p^{sda^{*}} \right|-w^{sda^{*}}=\frac{\left( 1+q-\eta\mu+s_{g} \right)\left( \alpha_{d}^{2}-\alpha_{c}\left( 1-\alpha_{d} \right) \right)}{1-\left( \alpha_{c}+\alpha_{d} \right)^{2}}$, $0<\alpha_{c}<1-\alpha_{d}$ by Proposition 7, therefore if $\alpha_{d}>\frac{1}{2}$,$\left| p^{sda^{*}} \right|-w^{sda^{*}}>\frac{\left( 1+q-\eta\mu\right)\left( 2\alpha_{d}-1 \right)}{1-\left( \alpha_{c}+\alpha_{d} \right)^{2}}>0$.

(4) Subsidizing S&T innovation platform. $\left| p^{spa^{*}} \right|-w^{spa^{*}}=\frac{\left( 1+q-\eta\mu\right)\left( \alpha_{d}\left( \alpha_{d}+s_{g} \right)-\left( \alpha_{c}+s_{g} \right)\left( 1-\alpha_{d}-s_{g} \right) \right)}{1-\left( \alpha_{c}+\alpha_{d}+s_{g} \right)^{2}}$, $0<\alpha_{c}<1-\alpha_{d}-s_{g}$ by Proposition 10, and therefore if $\alpha_{d}>\frac{1-s_{g}}{2}$时,$\left| p^{spa^{*}} \right|-w^{spa^{*}}>0$.

The proof is completed.

## A.20 Proof of Property 8

(1) The S&T innovation platform charges research users:$p^{c^{*}}>0$, $p^{scc^{*}}>0$, if $\alpha_{c}>\alpha_{d}$,$p^{sdc^{*}}>0$, if $\alpha_{c}<\alpha_{d}$ and $s_{g}>\frac{\left( 1+q-\eta\mu\right)\left( 2-\alpha_{d}\left( \alpha_{c}+\alpha_{d} \right) \right)}{\alpha_{d}-\alpha_{c}}$, $p^{sdc^{*}}<0$; if $0<\alpha_{c}<\frac{2-\left( \alpha_{d}+s_{g} \right)^{2}}{\alpha_{d}+s_{g}}$,$p^{spc^{*}}>0$，if $\alpha_{c}>\frac{2-\left( \alpha_{d}+s_{g} \right)^{2}}{\alpha_{d}+s_{g}}$, $p^{spc^{*}}<0$.

$p^{sdc^{*}}-p^{c^{*}}=\frac{s_{g}\left( \alpha_{c}-\alpha_{d} \right)}{4-\left( \alpha_{c}+\alpha_{d} \right)^{2}}$, if $\alpha_{c}>\alpha_{d}$, $p^{sdc^{*}}>p^{c^{*}}$, if $\alpha_{c}<\alpha_{d}$, $p^{sdc^{*}}<p^{c^{*}}$,

$p^{scc^{*}}-p^{sdc^{*}}=\frac{s_{g}\left( 2-\alpha_{c}+\alpha_{d}\left( 1-\alpha_{c}-\alpha_{d} \right) \right)}{4-\left( \alpha_{c}+\alpha_{d} \right)^{2}}>0$,

$p^{scc^{*}}-p^{spc^{*}}=\frac{s_{g}\left( \left( 1+q-\eta\mu\right)\left( 2\left( s_{g}+2\alpha_{d} \right)-\alpha_{c}\left( \alpha_{c}+\alpha_{d} \right)\left( \alpha_{c}+\alpha_{d}+s_{g} \right) \right)+\left( 2-\alpha_{d}\left( \alpha_{c}+\alpha_{d} \right) \right)\left( 4-\left( \alpha_{c}+\alpha_{d}+s_{g} \right)^{2} \right) \right)}{\left( 4-\left( \alpha_{c}+\alpha_{d} \right)^{2} \right)\left( 4-\left( \alpha_{c}+\alpha_{d}+s_{g} \right)^{2} \right)}>0$,

$p^{sdc^{*}}-p^{spc^{*}}=\frac{s_{g}\left( \left( 1+q-\eta\mu\right)\left( 2\alpha_{d}+s_{g} \right)\left( 2-\alpha_{c}\left( \alpha_{c}+\alpha_{d} \right) \right)+\left( \alpha_{c}-\alpha_{d} \right)\left( 4-\left( \alpha_{c}+\alpha_{d}+s_{g} \right)^{2} \right) \right)}{\left( 4-\left( \alpha_{c}+\alpha_{d} \right)^{2} \right)\left( 4-\left( \alpha_{c}+\alpha_{d}+s_{g} \right)^{2} \right)}>0$,

$p^{c^{*}}-p^{spc^{*}}=\frac{s_{g}\left( 1+q-\eta\mu\right)\left( \left( \alpha_{d}+s_{g} \right)\left( 4-\left( \alpha_{c}+\alpha_{d} \right)^{2} \right)-s_{g}\left( 2-\alpha_{d}\left( \alpha_{c}+\alpha_{d} \right) \right) \right)}{\left( 4-\left( \alpha_{c}+\alpha_{d} \right)^{2} \right)\left( 4-\left( \alpha_{c}+\alpha_{d}+s_{g} \right)^{2} \right)}>0$,

$p^{scc^{*}}-p^{c^{*}}=\frac{s_{g}\left( 2-\alpha_{d}\left( \alpha_{c}+\alpha_{d} \right) \right)}{4-\left( \alpha_{c}+\alpha_{d} \right)^{2}}>0$, therefor if $\alpha_{c}>\alpha_{d}$, $p^{scc^{*}}>p^{sdc^{*}}>p^{c^{*}}>p^{spc^{*}}$, if $\alpha_{c}<\alpha_{d}$, $p^{scc^{*}}>p^{c^{*}}>p^{sdc^{*}}>p^{spc^{*}}$.

(2) The S&T innovation platform pays commission rebates to providers: when $\alpha_{d}<\alpha_{c}$, $w^{c^{*}}>0$, $w^{scc^{*}}>0$, $w^{spc^{*}}>0$, where if $s_{g}<\frac{\left( 1+q-\eta\mu\right)\left( \alpha_{c}-\alpha_{d} \right)}{2-\alpha_{c}\left( \alpha_{c}+\alpha_{d} \right)}$, then $w^{sdc^{*}}>0$, if $s_{g}>\frac{\left( 1+q-\eta\mu\right)\left( \alpha_{c}-\alpha_{d} \right)}{2-\alpha_{c}\left( \alpha_{c}+\alpha_{d} \right)}$, then $w^{sdc^{*}}<0$；when $\alpha_{d}>\alpha_{c}$，$w^{c^{*}}<0$，$w^{scc^{*}}<0$，$w^{sdc^{*}}<0$，where if $s_{g}<\alpha_{d}-\alpha_{c}$,then $w^{spc^{*}}<0$；where if $s_{g}>\alpha_{d}-\alpha_{c}$，then $w^{spc^{*}}>0$. $w^{sdc^{*}}-w^{c^{*}}=\frac{-s_{g}\left( 2-\alpha_{c}\left( \alpha_{c}+\alpha_{d} \right) \right)}{4-\left( \alpha_{c}+\alpha_{d} \right)^{2}}<0$，$w^{scc^{*}}-w^{{sdc}^{*}}=\frac{s_{g}\left( \alpha_{c}\left( 1-\alpha_{c}-\alpha_{d} \right)+2-\alpha_{d} \right)}{4-\left( \alpha_{c}+\alpha_{d} \right)^{2}}>0$，$w^{scc^{*}}-w^{c^{*}}=\frac{s_{g}\left( \alpha_{c}-\alpha_{d} \right)}{4-\left( \alpha_{c}+\alpha_{d} \right)^{2}}$，

$w^{spc^{*}}-w^{c^{*}}=\frac{s_{g}\left( 1+q-\eta\mu\right)\left( \left( \alpha_{c}-\alpha_{d} \right)\left( 2\left( \alpha_{c}+\alpha_{d} \right)+s_{g} \right)+4-\left( \alpha_{c}+\alpha_{d} \right)^{2} \right)}{\left( 4-\left( \alpha_{c}+\alpha_{d} \right)^{2} \right)\left( 4-\left( \alpha_{c}+\alpha_{d}+s_{g} \right)^{2} \right)}$，

$w^{spc^{*}}-w^{scc^{*}}=\frac{s_{g}\left( \left( 1+q-\eta\mu\right)\left( 2\left( 2-\alpha_{d}\left( \alpha_{c}+\alpha_{d} \right) \right)+\left( \alpha_{c}-\alpha_{d} \right)\left( \alpha_{c}+\alpha_{d}+s_{g} \right) \right)-\left( \alpha_{c}-\alpha_{d} \right)\left( 4-\left( \alpha_{c}+\alpha_{d}+s_{g} \right)^{2} \right) \right)}{\left( 4-\left( \alpha_{c}+\alpha_{d} \right)^{2} \right)\left( 4-\left( \alpha_{c}+\alpha_{d}+s_{g} \right)^{2} \right)}$if $\alpha_{c}<\alpha_{d}$，$w^{sp{}^{*}}>w^{scc^{*}}$，$w^{scc^{*}}<w^{c^{*}}$，$w^{spc^{*}}<w^{c^{*}}$,therefore $w^{c^{*}}>w^{spc^{*}}>w^{scc^{*}}>w^{{sdc}^{*}}$.

The proof is completed.
